# Functional, Biophysical, and Structural Characterization of Human IgG1 and IgG4 Fc Variants with Ablated Immune Functionality

**DOI:** 10.3390/antib6030012

**Published:** 2017-09-01

**Authors:** Susan H. Tam, Stephen G. McCarthy, Anthony A. Armstrong, Sandeep Somani, Sheng-Jiun Wu, Xuesong Liu, Alexis Gervais, Robin Ernst, Dorina Saro, Rose Decker, Jinquan Luo, Gary L. Gilliland, Mark L. Chiu, Bernard J. Scallon

**Affiliations:** Janssen Research & Development, LLC, 1400 McKean Road, Spring House, Ambler, PA 19477, USA; aarmst12@its.jnj.com (A.A.A.); ssomani@its.jnj.com (S.S.); swu4@its.jnj.com (S.-J. W.); xliu8@its.jnj.com (X.L.); agervais@its.jnj.com (A.G.); rernst2@its.jnj.com (R.E.); dsaro@its.jnj.com (D.S.); rdecker5@its.jnj.com (R.D.); jluo@its.jnj.com (J.L.); ggillila@its.jnj.com (G.L.G.); bhscallon@gmail.com (B.J.S.)

**Keywords:** Fc engineering, silent effector function, IgG1, IgG2, IgG4, IgG sigma, developability, pharmacokinetics, crystal structure

## Abstract

Engineering of fragment crystallizable (Fc) domains of therapeutic immunoglobulin (IgG) antibodies to eliminate their immune effector functions while retaining other Fc characteristics has numerous applications, including blocking antigens on Fc gamma (Fcγ) receptor-expressing immune cells. We previously reported on a human IgG2 variant termed IgG2σ with barely detectable activity in antibody-dependent cellular cytotoxicity, phagocytosis, complement activity, and Fcγ receptor binding assays. Here, we extend that work to IgG1 and IgG4 antibodies, alternative subtypes which may offer advantages over IgG2 antibodies. In several in vitro and in vivo assays, the IgG1σ and IgG4σ variants showed equal or even lower Fc-related activities than the corresponding IgG2σ variant. In particular, IgG1σ and IgG4σ variants demonstrate complete lack of effector function as measured by antibody-dependent cellular cytotoxicity, complement-dependent cytotoxicity, antibody-dependent cellular phagocytosis, and in vivo T-cell activation. The IgG1σ and IgG4σ variants showed acceptable solubility and stability, and typical human IgG1 pharmacokinetic profiles in human FcRn-transgenic mice and cynomolgus monkeys. In silico T-cell epitope analyses predict a lack of immunogenicity in humans. Finally, crystal structures and simulations of the IgG1σ and IgG4σ Fc domains can explain the lack of Fc-mediated immune functions. These variants show promise for use in those therapeutic antibodies and Fc fusions for which the Fc domain should be immunologically “silent”.

## 1. Introduction

The biological functionality of therapeutic antibodies depends on the interactions of two regions of the protein with components of its external environment: the antigen binding region (Fab) interacting with an antigen, and the fragment crystallizable (Fc) region interacting with components of the immune system. The Fc region of the immunoglobulin (IgG) antibody, which is the focus of this study, can have interactions with Fc gamma receptors (FcγR) and first subcomponent of the C1 complex (C1q) that mediate antibody-dependent cellular cytotoxicity (ADCC), complement-dependent cytotoxicity (CDC), antibody-dependent cellular phagocytosis (ADCP), induction of secretion of mediators, endocytosis of opsonized particles, as well as modulation of tissue and serum half-life through interaction with the neonatal Fc receptor (FcRn) [1,2]. Numerous publications have reviewed the application of enhanced Fc effector function to increase biologic activity [3,4,5,6]. In addition, the coupling of the Fab and Fc regions can impact the therapeutic window for safety and efficacy of antibodies and Fc fusion proteins [3,7].

Fc-mediated effector functions are best avoided for some applications such as systemic neutralization of cytokines, targeting cell surface antigens on immune cells, or when engineering bispecific molecules to bring target diseased cells within proximity of effector immune cells to provide a more specific immune receptor engagement [3,8,9]. In each of these cases, it is best not to stimulate unwanted cell and tissue damage or risk undesired effector cell activation, immune cell depletion, or FcγR cross-linking that might induce cytokine release through engagement of Fc-mediated effector functions [3]. An important consideration in such biological processes is that the complexity of FcγR functional properties is increased by the varying densities of activating and inhibitory receptors on the different effector cell populations [10]. Likewise, since the threshold of activation can be variable with different patients, it would be prudent for safety considerations to develop antibodies with a more silent Fc framework. Thus, development of completely silent Fc domains can be critical for biologics that do not require FcγR or C1q mediated effector functions [11].

When an antibody with no effector function is required, there are different approaches one may take to generate a molecule with the desired properties. Unfortunately, results of some strategies often come with liabilities to the molecular profile. For instance, Fab or F(ab′)_2_ fragments can be generated; however, such molecules have shorter half-lives in patient sera. Chemical modifications can extend the half-life of such molecules, but can also bring potential risks with toxicities [12]. Another strategy has been to eliminate the N-linked glycosylation at residue Asparagine 297 (European Union (EU) numbering) [13,14,15,16]; however, this can reduce antibody solubility and stability. Another approach employs mutagenesis of specific Fc amino acid residues to specifically influence effector functions [17].

An example of this approach is illustrated in the first marketed therapeutic antibody (Orthoclone OKT3, a murine IgG2a) in which two mutations in the lower hinge (L324A/L235A, referred to as AA) were introduced to mitigate the induction of cytokine storm [18,19]. Also, because FcγRs are highly selective in subclass specificity and affinity [20,21], another approach may be to move the Fab domains onto Fc regions which elicits less effector function such as human IgG2 (huIgG2) or IgG4 (huIgG4) [22]. In addition, swapping among the sequences of the four human IgG (huIgG) subtypes has been used to design more silent Fc domains [3,4,23,24,25] that have resulted in such variants as huIgG2/4 [26], huIgG2m4 [27], and L234F/L235E/P331S (FES) [28]. Notably, Vafa and co-workers employed multiple strategies to develop a huIgG2 variant, termed huIgG2 sigma (IgG2σ) that showed undetectable Fc-mediated effector function and C1q binding [29]. In utilizing such strategies for silencing Fc effector function, it needs to be recognized that there is some potential for huIgG2 subtype molecules to form heterogeneous isoforms which can be a challenge in the generation of a homogeneous product [30,31,32].

Although huIgG4 has weak binding affinity to most FcγRs except for the high affinity receptor FcγRI, it does retain the ability to induce phagocytosis by macrophages (expressing FcγRI, FcγRIIa, and FcγRIIIa) and possibly activate monocytes when in an immune complex due to activating FcγRs on specific immune cells. Recent additional approaches to generate antibodies with no effector function have included disruption of proline sandwich motifs [33], and incorporation of asymmetric charged mutations in the lower hinge or constant heavy chain domain 2 (C_H_2) domain [34]. Because development of antibodies with silent Fc domains continues to be important for various therapies, and because the threshold of activation may be different for each patient or disease population, efforts are on-going to obtain the most silent Fc variants which will have improved safety and good manufacturing qualities.

We describe here the functional and structural characteristics of three novel silent Fc designs: huIgG1 sigma (IgG1σ), which is a variant of huIgG1, and huIgG4 sigma1 (IgG4σ1) and huIgG4 sigma2 (IgG4σ2), which are variants of huIgG4. The effector functions of these silent Fc variants are compared to those of previously described constructs such as huIgG1 L234A/L235A (AA) [25], huIgG4 S228P/L234A/L235A (PAA) [19,35], and huIgG1 L234F/L235E/P331S (FES, a triple mutant being employed in a clinical anti-interferon receptor antibody [36]). We also present a comparison of serum half-lives in mice and cynomolgus monkeys, an evaluation of potential immunogenicity, and an assessment of biophysical stability. Crystal structures and molecular modeling were carried out to understand the mechanism for lack of interactions between the IgG variants and the Fc gamma receptors. The aim of the studies presented here is to provide data on the biological, biophysical, and structural properties of the huIgG1σ, huIgG4σ1, and huIgG4σ2 along with other commonly used silent Fc formats to enable development of better quality antibody therapeutics.

## 2. Results

### 2.1. Design of Silent Fc HuIgGs

**Figure 1:**
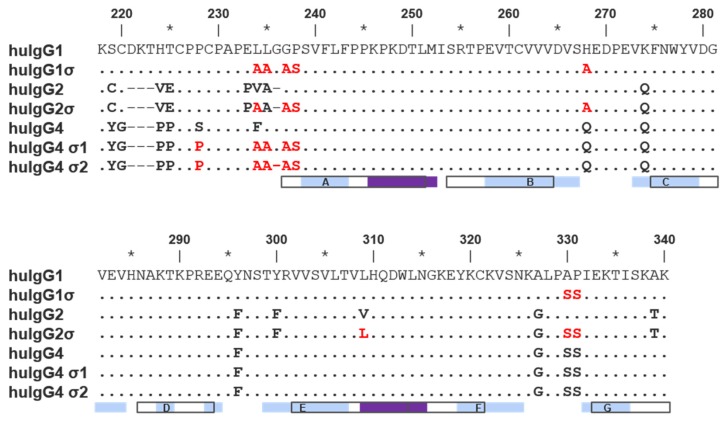
Alignment of hinge and constant heavy chain domain 2 (C_H_2) domain amino acid sequences of wild-type human immunoglobulin G1 (IgG1), IgG2 and IgG4 as well as their sigma variants. The alignment above uses EU numbering. Residues identical to wild-type IgG1 are indicated as dots; gaps are indicated with hyphens. Sequence is given explicitly if it differs from wild-type IgG1 or from the parental subtype for σ variants. In the latter case, sequence is colored red. Open boxes beneath the alignment correspond to International Immunogenetics Information System (IMGT) strand definitions (labeled) [39]. Light blue and purple boxes beneath the alignment correspond to the strand and helix secondary structure assignment for wild-type IgG1 in the Protein Data Bank (PDB) 3AVE (chain A) [40]. Residues 267–273 form the BC loop and 322–332 form the FG loop.

Silent human IgG Fcs were designed by uniquely combining the sequence from previously characterized variants having mutations in the hinge and C_H_2 regions [29]. Mutations present in these variants are listed in Figure 1 and Table 1. All sequences described follow the EU numbering system [37,38].

**Figure 2:**
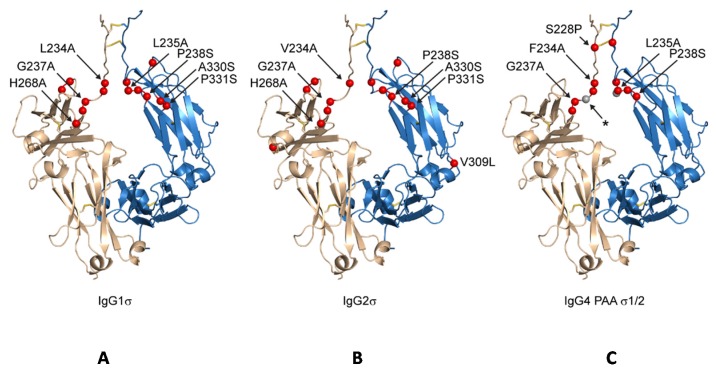
Schematic indicating positions of mutations on generic Fc structure. Positions mutated relative to parental subtype are depicted as red spheres on a structure of IgG1 Fc having an idealized hinge (wheat and blue, cartoon) for IgG1σ (**A**), IgG2σ (**B**), and IgG4σ1/2 (**C**). For clarity, the sites of mutation are labeled on only one of two equivalent Fc chains. IgG4σ2 differs from IgG4σ1 by the deletion of G236 (position indicated with a small, grey sphere and labeled with an asterisk).

Both huIgG1σ and the huIgG2σ constructs contain a total of seven mutations in their hinge and C_H_2 domains (Figure 1 and Figure 2; Table 1). However, only six of the mutations are common to both huIgG2σ and the huIgG1σ variant described here. HuIgG1σ uniquely includes an L234A mutation, and huIgG2σ has a V309L mutation. HuIgG1 naturally has a leucine at that position.

Compared to huIgG4 PAA, huIgG4σ1 and huIgG4σ2 have two and three additional mutations, respectively, for a total of five and six mutations (Table 1). While modeled after huIgG2σ, these huIgG4 variants do not have the full set of sigma mutations present in huIgG2σ because the huIgG4 sequence naturally contains four of those residues.

### 2.2. FcγR Binding and Competition

Since effector functions such as ADCC and ADCP are induced by the binding of antibody Fc with the various FcγRs on the surface of different immune cell types, it is crucial to minimize and if possible to eliminate this engagement. Testing for binding of the assorted Fc variants to human FcγRs was determined using an AlphaScreen competitive binding assay. The AlphaScreen competition strategy involves the following: biotin-labeled huIgG is captured on streptavidin donor beads; His-tagged FcγRs are captured on nickel acceptor beads; unlabeled competitor (test) antibodies are applied as serial dilutions. There is a reduction in (maximal) signal when competition takes place. To provide an avidity component comparable to that created by multiple antibodies bound to a cell surface, the test antibodies were cross-linked with anti-F(ab′)_2_. This method increases the sensitivity of the assay to aid in confirming low-level binding.

**Figure 3:**
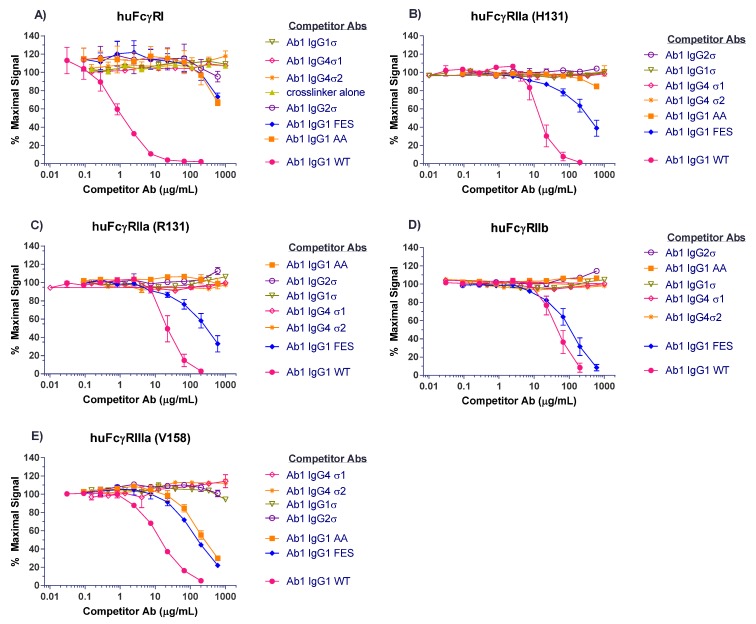
Testing for interactions of cross-linked huIgG variants with Fcγ receptors. Binding of huIgG Ab1 (anti-TNFα) molecules to human FcγRs was assessed using AlphaScreen bead assays in a competitive format. To increase sensitivity of the assays, test samples were cross-linked to introduce binding avidity by using a goat F(ab′)_2_ anti-huIgG F(ab′)_2_-specific fragment in 1:1 molar ratio with the test antibodies. Cross-linked test antibodies at the designated concentrations were co-incubated with biotin-labeled huIgG Fc fragment (to avoid binding to cross-linker), the respective His-tagged FcγRs, nickel chelate acceptor beads, and streptavidin donor beads. Plates were read on the EnVision multi-label plate reader, and data plotted with GraphPad Prism v6.0 software. Shown are binding of cross-linked huIgG variants to: (**A**) huFcγRI, (**B**) huFcγRIIa-H131, high affinity allotype, (**C**) huFcγRIIa-R131, low affinity allotype, (**D**) huFcγRIIb, (**E**) huFcγRIIIa-V158, high affinity allotype. Non-binding of cross-linker alone is shown with the high affinity huFcγRI. All points represent the mean of duplicate samples ± range. The plot labels refer to IgG1 AA—human IgG1 L234A/L235A; IgG1 FES—human IgG1 L234F/L235E/P331S; and IgG1 WT—wild type human IgG1.

Competitive binding analyses of Ab1 (anti-tumor necrosis factor (TNF)α huIgG1) samples to the different FcγRs are shown in Figure 3. On CD64 receptor (FcγRI), Ab1 huIgG1 wild-type (WT) binds well with 50% inhibition at approximately 1 μg/mL. HuIgG1 AA and huIgG1 FES show low level binding at 500 μg/mL, the highest concentration tested. However, the huIgG1σ, huIgG4σ1, and huIgG4σ2 variants show no indications of binding, even at concentrations up to 1 mg/mL (Figure 3A). On CD32a (huFcγRIIa-H131 high and huFcγRIIa-R131 low affinity variants), CD32b (huFcγRIIb), and CD16a (huFcγRIIIa), the huIgG1 AA and huIgG1 FES variants showed varying degrees of binding to these receptors, yet the sigma (σ) variants showed complete lack of binding compared to the huIgG1 wild-type (WT) (Figure 3B–E). HuIgG4 PAA was not included in this test panel due to reagent constraints, but huIgG4 PAA has been reported to have reduced, but not eliminated binding to FcγRs [29,33]. These collective data demonstrated that complexes formed from the IgG1σ and IgG4σ variants did not bind to FcγRs even at high concentrations.

### 2.3. Target Mediated Effector Function In Vitro

#### 2.3.1. Antibody-Dependent Cell-Mediated Cytotoxicity (ADCC)

**Figure 4:**
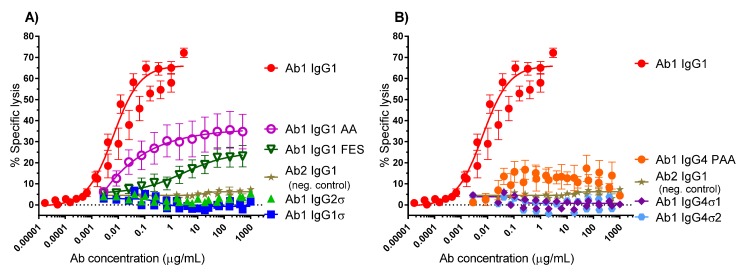
Antibody-dependent cellular cytotoxicity (ADCC) assays (combined data from six experiments). Titrating amounts of test antibodies (Abs) were added to K2 target cells, followed by addition of human PBMC immune effector cells. The extent of target cell lysis was quantitated after 2 h. Samples were tested in duplicate for each individual experiment. In the combined data shown here, 15 donors were tested, and each point represents the mean value ± standard error of the mean. % Specific lysis is shown in (**A**) for IgG1σ compared to IgG1 AA, FES, and IgG2σ; and in (**B**) for IgG4σ1 and IgG4σ2 compared to IgG4 PAA (S228P/F234A/L235A). Ab1 IgG was used as the positive control and Ab2 IgG1 (neg. control) was the negative control.

When the Fc region of an antibody binds to FcγR on immune cells, cytotoxic factors are released causing ADCC and death of target cells, whose membrane-surface antigens have been bound by the specific antibody. To compare cytotoxic potential of the silent Fc variants, K2 cells (T72-18, TNFα SP2/0) expressing the Δ1–12 variant of human TNFα were used as target cells and human peripheral mononuclear cells (PBMCs) were the immune effector cells in ADCC assays [41]. Figure 4 shows the combined data from six independent experiments that used PBMC effector cells from 15 different donors. Figure 4A shows that Ab1 huIgG1σ and huIgG2σ have no cytotoxic activity compared to huIgG1 AA, huIgG1 FES variants and the positive control Ab1 huIgG1 WT. Figure 4B shows also that Ab1 huIgG4σ1 and huIgG4σ2 have no cytotoxic activity compared to the huIgG4 PAA and huIgG1. At concentrations up to 1 mg/mL, Ab1 huIgG1σ, huIgG2σ, huIgG4σ1 and huIgG4σ2 showed minimal to no killing of target cells. In particular, the sigma variants displayed negligible cytotoxic activity at levels below that of the negative control Ab2 (anti-F glycoprotein of Respiratory Syncytial Virus a huIgG1) which did not bind to TNFα. In this assay with numerous donors, Ab1 huIgG1 AA was the least silent Fc variant; HuIgG1 AA, huIgG1 FES, and huIgG4 PAA showed intermediate cytotoxic levels, and the most ADCC-silent molecules were the huIgG1σ and huIgG4σ variants.

#### 2.3.2. Antibody-Dependent Cellular Phagocytosis (ADCP)

**Figure 5:**
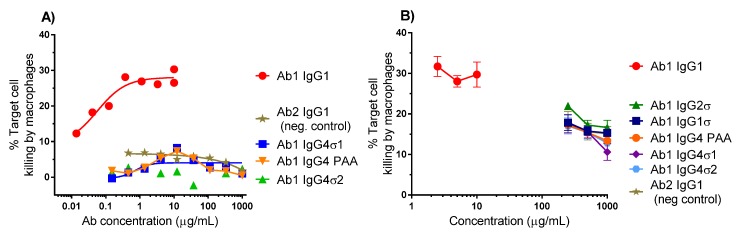
Antibody-Dependent Cellular Phagocytosis (ADCP) (phagocytosis) assays. The extent of phagocytosis was measured using flow cytometry. (**A**) A representative experiment, showing the %Target cell killing by macrophages in a 5 h assay with different concentrations of test antibodies; (**B**) Results using macrophages from a different donor tested in triplicate with a focus on high Ab concentrations (200–1000 μg/mL). % Target cell killing is the number of dual-labeled cells (target cells engulfed by macrophages) divided by total number of target cells × 100. Error bars represent ± standard error of the mean.

Binding of the Fc region to FcγRs on certain immune cells such as macrophages can cause cell death by phagocytosis. ADCP relies on macrophages to bind and devour target cells following antibody binding. To further evaluate the lack of effector function with the silent Fc variants, ADCP flow cytometric assays were performed using macrophages derived from primary human monocytes as effector cells and K2 cells as target cells. Macrophages and K2 cells were labeled with separate fluorescent dyes and co-cultured for 5 h in the presence of different concentrations of test antibody. Quantitation of antibody-opsonized target cells was measured by dual-label flow cytometry. Representative data from two experiments are shown in Figure 5. Ab1 huIgG1 shows approximately 30% target cell killing by macrophages, whereas Ab1 huIgG4 PAA, Ab1 huIgG4σ1, and Ab1 huIgG4σ2 show low level killing (<5%), comparable with that of Ab2 (non-TNF binding) IgG1 (Figure 5A). In another study, Ab huIgG1 shows a similar extent of cell killing by macrophages (using a different donor), but Ab1 huIgG4σ1, Ab1 huIgG4σ2 and Ab1 huIgG1σ1 (along with Ab1 huIgG2σ, Ab1 huIgG4 PAA and Ab2 huIgG1 negative control) show minimal K2 target cell phagocytosis relative to positive and negative controls at concentrations up to 1 mg/mL (Figure 5B). These results suggest that the huIgG1σ and huIgG4σ variants would not have Fc-mediated cytotoxic potential when bound to target immune cells.

#### 2.3.3. Complement-Dependent Cytotoxicity (CDC)

**Figure 6:**
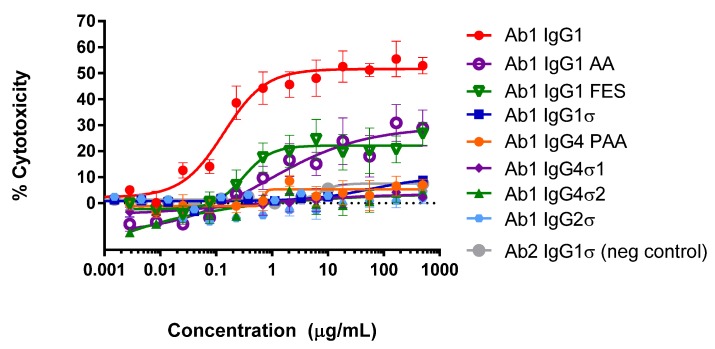
Cell-based CDC assays with IgG Fc variants. K2 mouse myeloma cells stably expressing a transmembrane form of human tumor necrosis factor alpha (TNFα) were incubated with varying concentrations of test antibody and rabbit complement, and then cell viability was measured by detecting lactate dehydrogenase release in the culture supernatant. Error bars represent standard error of the mean from samples analyzed in triplicate. Ab2 IgG1σ does not bind to target cells and is the negative control antibody.

Complement-dependent cell killing occurs when the Fc portion recruits serum complement proteins to the cell bound by the specific antibody; leading to induction of a membrane attack complex and target cell lysis. To determine whether the Fc variants have complement activation capability, CDC assays were performed using K2 target cells, rabbit complement and a panel of Ab1 huIgG Fc variants. As shown in Figure 6 using Ab1 variants, CDC results indicate that huIgG1σ, huIgG4 PAA, huIgG4σ1 and huIgG4σ2, and huIgG2σ samples have minimal activity, like that of the negative control Ab2 huIgG1 at concentrations up to 500 μg/mL. In contrast to the negative control, the positive control Ab1 huIgG1 has measurable target mediated specific killing activity at less than 0.1 μg/mL. Relative to these controls, Ab1 huIgG1 AA and huIgG1 FES show intermediate levels of cytotoxicity. Data indicate that Ab1 huIgG1σ, huIgG4 PAA, huIgG4σ1, huIgG4σ2 and huIgG2σ lack specific CDC-inducing activity.

### 2.4. FcRn Binding

**Figure 7:**
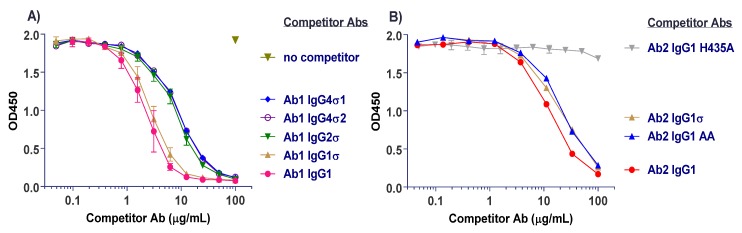
Binding of IgG variants to neonatal Fc receptor (FcRn). In a competition binding format, titrating amounts of antibody were combined with a fixed concentration (4 μg/mL) of biotin-labeled huIgG1. Antibody mixtures were incubated with immobilized human FcRn on a plate for 1 h, washed, detected with streptavidin-horseradish peroxidase (HRP), and developed with tetramethylbenzidine (TMB) substrate. Bound tracer antibody was detected via absorbance at 450 nm measured on a plate reader. (**A**) Ab1 samples and (**B**) Ab2 samples were tested in duplicate and error bars represent mean ± range.

Interaction with neonatal Fc receptor (FcRn) is a critical factor that contributes to sustaining circulating antibody half-life [2], thus better binding to FcRn is expected to give a longer antibody half-life. To determine whether the sigma Fc mutations affect FcRn binding, competition binding by an enzyme-linked immunosorbent assay (ELISA) was performed with varying concentrations of unlabeled silent Fc antibody and a fixed amount of biotin-labeled huIgG1 on plate-bound human FcRn at pH 6. Bound tracer antibody was determined by colorimetric detection after incubation with streptavidin-horseradish peroxidase (HRP) and tetramethylbenzidine (TMB) substrate reagents. ELISA results shown in Figure 7 indicate a slight (less than 2-fold) reduction in binding affinity to FcRn for huIgG1σ relative to the huIgG1 wild-type control. Other tested variants show up to 4-fold reductions in relative affinity. The negative control variant (huIgG1 H435A) of Ab2 appropriately shows no binding to FcRn. While these findings indicate some reduction in relative affinity for the variants tested, all the silent Fc variants can bind FcRn, and huIgG1σ appears to bind FcRn the best.

### 2.5. FcγR Binding Interactions In Vivo

#### 2.5.1. T Cell Immunostimulatory Activity

In vivo, the Fc and FcγR interaction can be evaluated and detected by T cell activation [42]. Human FcγR transgenic mice (FcγR-hu) express all five human FcγRs and have no expression of endogenous mouse FcγRs [43]. When anti-mouse CD3 antibody (Ab4, 2C11) binds to mouse T cells, there is an agonistic effect (presumably due to binding of FcγR on neighboring cells at the same time it is bound to CD3 on T cells) which leads to up-regulation of activation markers such as CD69 and CD25 [42]. An increase in binding of Fc to FcγR correlates with an increased level of T cell activation. This agonist activity was used to evaluate T cell activation of the Fc variants in FcγR-hu mice.

**Figure 8:**
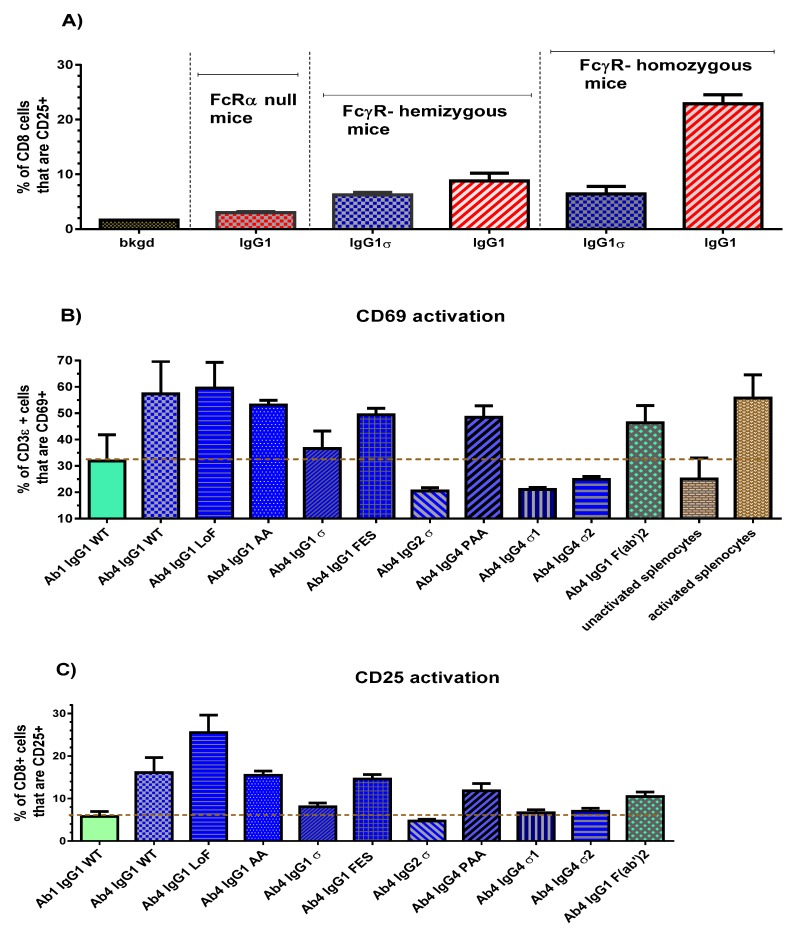
In vivo T cell activation. (**A**) Results of in vivo T cell activation by IgG1σ relative to wild-type IgG1. FcγR-humanized mice, homozygous and hemizygous, and mice lacking FcγRs (null) were injected intraperitoneal with a 0.5 mg/kg dose of either human IgG1σ or wild-type IgG1. After 24 h, spleens were collected from each mouse and the percent of CD8+ cells (as well as CD3ε cells, not shown) that stained positive for CD25 was measured by flow cytometric analyses. (**B**) Comparison of in vivo T cell CD69 activation with panel of silent IgG Fc variants. (**C**) Comparison of in vivo T cell CD25 activation with panel of silent IgG Fc variants. The dashed line represents the level of non-specific activation, derived from the mean value of un-activated splenocytes and splenocytes treated with a control IgG1 WT. Error bars represent the standard error of mean from 3 to 6 samples in both studies.

An initial study was done to confirm that T cell activation in the FcγR-hu mice is due to FcγR-Fc binding. Three strains of mice with different gene dosages were used: the 5-transgene FcγR-humanized mice, mice hemizygous for the 5 transgenes, and FcRα chain null mice (FcγR knockouts). Ab4 huIgG1 and Ab4 huIgG1σ were injected into the intraperitoneal cavity (0.5 mg/kg, 10 mL/kg). Approximately 24 h later, mice were euthanized and their spleens removed for T cell activation analyses by flow cytometry. Results demonstrated the dependence on the presence of human FcγR, since very little or no T cell activation using huIgG1 was detected in the null mice, an intermediate level of activation was detected in hemizygous mice, and the most robust activation was noted in the homozygous mice (Figure 8A). Low to no activation was observed in T cells of huIgG1σ treated mice. Next, homozygous FcγR-humanized mice were used to explore a panel of Ab4 silent Fc variants. Twenty-four hours following the dose of the test antibodies, splenocytes from the mice were isolated and flow analyses performed to determine T cell levels of the early activation marker (CD69), and a late activation marker (CD25).

Comparison of CD69 activation results (expressed as a percentage of CD3ε cells) are shown in Figure 8B for the silent Fc variants, a non-FcγR binding F(ab′)_2_ fragment and a non-T cell binding Ab1 huIgG1. Splenocytes from wild-type IgG (Ab4 huIgG1), low fucose IgG1 (Ab4 huG1 LoF with high affinity binding to CD16), and phorbol myristate acetate treated mice, collectively showed a robust CD69 signal (mean value 58%) compared to un-activated splenocytes (25%) from phosphate- buffered saline (PBS) treated mice. Ab4 F(ab′)_2_ gave a substantial unexpected signal (46%) which suggested that T cells may have a low level of activation merely by bivalent CD3 binding without FcγR engagement. Ab4 huIgG1 induced a higher CD69 activation level than Ab1 huIgG1 WT because it has both CD3 and FcγR engagement. The dashed line in Figure 8B represents a “non-specific activation” level (29%), determined from the average value of untreated splenocytes and splenocytes treated with a non-T cell binding Ab1 huIgG1. Ab4 huIgG1 AA, Ab4 huIgG1 FES and Ab4 huIgG4 PAA produced an increase in T cell activation relative to the non-specific activation level. However, Ab4 huIgG2σ, Ab4 huG4σ1 and Ab4 huG4σ2 caused a decrease in CD69 activation relative to the non-specific level, and Ab4 huIgG1σ caused an activation level like that of the Ab1 huIgG1 WT which does not bind T cells.

CD25 activation levels of splenic T cells (expressed as a percent of CD8+ cells) from mice treated with the silent Fc variants are shown in Figure 8C. The dashed line represents the level for non-specific activation derived from splenocytes treated with control Ab1 huG1 WT. Consistent with CD69 results, Ab4 huG1 AA, huG1 FES, and huG4 PAA produced an increase in CD25 activation levels that are comparable to that of Ab4 huG1 WT treated mice. As expected, the Ab4 huIgG1 LoF induced a higher CD25 activation signal. Likewise, Ab4 huG1 F(ab′)_2_ caused a higher CD25 activation than expected (such as in the case of CD69 activation) which may be due to bivalent CD3 binding, clustering, and signaling. Ab4 huIgG1σ, Ab4 huIgG2σ, Ab4 huG4σ1 and Ab4 huG4σ2 induced minimal CD25 activation. These in vivo CD69 and CD25 results indicated that binding of IgG1σ, IgG2σ, IgG4σ1 and IgG4σ2 variants did not activate T cells compared to T cells from mice treated with huIgG WT or huIgG4 PAA.

#### 2.5.2. FcγR-Mediated Anti-Tumor Activity

The B16 metastatic melanoma syngeneic model has been used to assess Fc and FcγR interactions. B16F10 mouse melanoma tumor cells can stimulate lung tumor metastasis, and murine TA99 antibody which targets the gp75 antigen on B16F10 cells, can inhibit tumor cell growth in mice [44,45]. Also, human TA99 antibody can inhibit metastasis, whereas a human IgG1 mutant which does not engage FcγRs has no effect in FcγR-hu mice [43].

**Figure 9:**
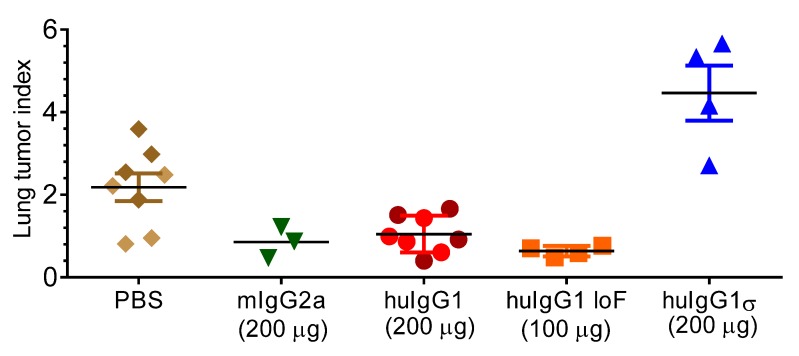
Effect of silent Fc mutations on syngeneic tumor cell metastasis. FcγR-humanized mice were injected IV with B16F10 cells and received either: phosphate-buffered saline (PBS), Ab5 (TA99) mIgG2a, Ab5 huIgG1, Ab5 huIgG1 LoF (low fucose) or Ab5 huIgG1σ (*n* = 4 for groups) on days 0, 2, 4, 7, 9 and 11. On day 21, lungs were harvested and metastasis foci were counted. Data show the mean number of lung metastasis foci expressed as lung tumor index from mice receiving the indicated treatment ± standard error.

This B16 metastatic tumor model was used to test the effect of a huIgG1σ version of Ab5 (anti-grp75) on lung tumor metastasis in FcγR-hu mice. These mice treated with different versions of Ab5: huIgG1, huIgG1 LoF (low fucose with enhanced ADCC, in-house data) and mIgG2a (mouse Ab as positive control) showed a significant reduction in lung tumor metastasis compared to the mice treated with PBS (Figure 9). Lung metastasis foci were decreased by 2-fold in mice treated with Ab5 mIgG2a or Ab5 huIgG1, and 3-fold with Ab5 huIgG1 LoF compared to the PBS treated mice. In contrast, mice treated with huIgG1σ showed no tumor inhibition, and although unexplained, huIgG1σ treated mice showed more tumor metastasis than the PBS treated mice. Results indicate that in vivo antibody-mediated tumor clearance appears to be dependent on binding to FcγRs by huIgG1 or huIgG1 LoF, whereas huIgG1σ which does not bind FcγR is unable to prevent tumor cell growth.

### 2.6. Pharmacokinetics (PK)

PK studies were done to determine whether normal antibody half-life would be altered using antibodies with silent sigma Fc mutations compared to the wild-type huIgG. Human FcRn transgenic mice (Tg32 hemi, 8–10 weeks old, 3–4 per group) were intravenously-injected once with 2 mg/kg doses of the test antibody. Blood was collected at various times up to 35 days post-injection and sera prepared by standard procedure. A Meso Scale Discovery (MSD) assay using anti-human IgG specific reagents was employed for quantitating human IgG in the mouse serum samples. Terminal half-life values were obtained using a one-compartment linear fit using Prism 6.0 software (GraphPad, San Diego, CA, USA).

**Figure 10:**
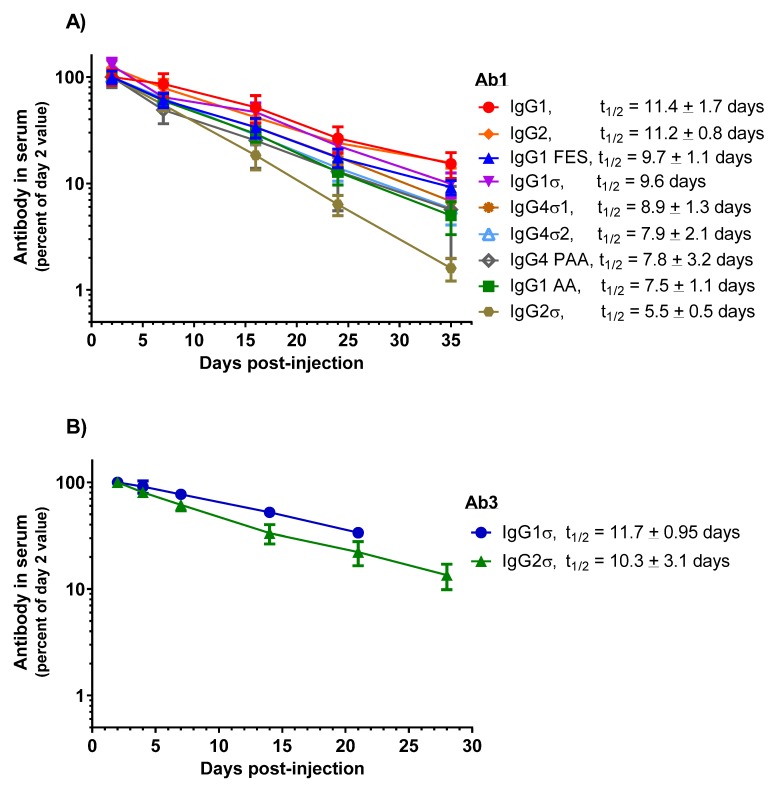
PK comparison of IgG1 WT and a panel of silent Fc variants in mice and cynomolgus monkeys. (**A**) Tg32 human FcRn transgenic mice were IV-injected with 2 mg/kg doses. Serum levels were expressed as a percent of day 2 values, the first timepoint. Data points represent the mean of 3 or 4 mice ± SEM. (**B**) Cynomolgus monkeys were injected intravenously with 1.5 mg/kg dose of an IgG2σ version of Ab3 bispecific antibody and the mean PK profile was compared (in a separate study) with monkeys injected with 1.5 mg/kg of an huIgG1σ version of Ab3. Similarly, IgG1σ and IgG2σ versions of Ab2 × Ab5 antibody were evaluated in the two separate studies.

HuIgG serum concentrations over time for the mice are shown in Figure 10A. PK profiles display a linear decrease in IgG levels from day 7 through 35. HuIgG2σ (t_1/2_ = 5.5 ± 0.5 days) shows a 2-fold shorter half-life than the huIgG1 (t_1/2_ = 11.4 ± 1.7 days). However, PK values for the other Fc variants: huIgG1 AA (t_1/2_ = 7.5 ± 1.1 days), huIgG4 PAA (t_1/2_ = 7.8 ± 3.2 days), huIgG4σ2 (t_1/2_ = 7.9 ± 2.1 days), huIgG4σ1 (t_1/2_ = 8.9 ± 1.3 days), huIgG1σ (t_1/2_ = 9.6 days), huIgG2 (t_1/2_ = 11.2 ± 0.8 days) and huIgG1 FES (t_1/2_ = 9.7 ± 1.1 days) are not significantly different from that of huIgG1. These results indicate that the silent Fc mutations do not alter the PK compared to normal huIgG1, except for huIgG2σ which showed a reduction in half-life.

PK studies in cynomolgus monkeys were conducted also to compare the huIgG1σ and huIgG2σ versions of Ab3, a bispecific antibody (anti-Respiratory Syncytial Virus (RSV)) and glycoprotein gp120 of HIV envelope, huIgG1). Male cynomolgus monkeys (2.5 to 3 kg, 3 per groups) were intravenously injected once with 1.5 mg/kg of the test antibody. Blood samples were collected up to 21 days or 28 days, and PK analyzed as described. Although performed in separate studies, similar half-life values are observed for Ab3 huIgG1σ (t_1/2_ = 11.7 ± 0.95 days) and its huIgG2σ counterpart (t_1/2_ = 10.3 ± 3.1 days); and for Ab2 × Ab5 bispecific (anti-RSV × anti-gp75) huIgG1σ (t_1/2_ = 5.6 ± 0.5 days) and its huIgG2σ counterpart (t_1/2_ = 5.2 ± 0.1 days) (Figure 10B). Considering that Ab2 huIgG1 historically has shown a half-life of 10–12 day in monkeys (Table S1), these monkey data along with the above mouse data, suggest that the huIgG1σ variant (as well as the huIgG2σ) should have a relatively normal serum half-life in humans.

### 2.7. Immunogenic Potential

**Figure 11:**
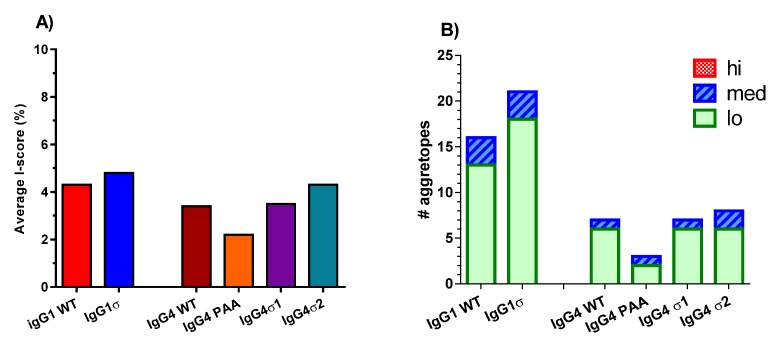
ImmunoFilter^TM^ analyses of the human IgG variants. The amino acid sequences for the different variants were analyzed for predicted immunogenicity *in silico* using ImmunoFilter^TM^ v2.7. (**A**) *IScores* summary which approximated the percent of US population expected to have a human leukocyte antigen (HLA) allele predicted to bind a nine-mer peptide within a sequence of interest. (**B**) The number of individual agretopes and their associated risk potential based on the percent of the population expected to have an allele predicted to bind each agretope (low (lo) = 10–25%, medium (med) = 25–50%, and high (hi) = over 50% of the U.S. population). Resulting *IScores* were averaged across all loci. Higher *IScores* indicate a higher predicted immunogenicity risk.

To assess the potential immunogenicity of huIgG1 and huIgG4 sigma variants, a T cell epitope analysis was done using the ImmunoFilter^TM^ v2.7 human leukocyte antigen (HLA) class II-peptide binding prediction software (Xencor, Inc., Monrovia, CA, USA) to predict potential immunogenicity in humans. These analyses generate *IScores*, which are weighted, population-relevant values that enable separation of individual 9-mer agretopes into groups by predicted immunogenic risk (PIR). Higher *IScores* indicate a higher PIR. Areas of sequence in the Fc variants which are identical to that of huIgG1 WT Fc sequence are excluded by the application of a tolerance threshold. *IScores* are averaged across all HLA-class II loci for each sequence of interest to create a single average *IScore* value. As shown in Figure 11A, the average *IScores* of the tested variant sequences are like those of their respective wild-type isotype controls. The greatest deviation from wild-type Fc results is a reduction of 1.2% observed with huIgG4 PAA. However, differences observed between the tested variants are inconsequential because all produce average *IScores* of less than 5%. *IScores* of less than 10% are considered to indicate very low risk of immunogenicity with a prediction that less than 10% of the U.S. population would have at least one allele predicted to bind a 9-mer located within the tested sequence. A further breakdown of the *IScore* data into number of predicted agretopes, as well as number of low, medium and high PIR agretopes for each sequence of interest is shown in Figure 11B. Again, individual predicted agretopes show all sequences having similar immunogenic risk profiles as their corresponding wild-type Fc sequences, where predicted agretopes would likely be tolerogenic. None of the Fc variants are predicted to generate any high PIR agretopes (i.e., over 50% of the U.S. population having at least one MHC allele predicted to bind the 9-mer).

### 2.8. Developability Assessment

A panel of biophysical assays was conducted on Abs with Fc variations to assess potential problems in developability that might impact the development and commercial manufacturing of therapeutics. These studies included assessing antibody thermal stability, the stability of highly concentrated samples over time, and stability at low pH. The results are described in the sub-sections below.

#### 2.8.1. Thermal Stability

Differential Scanning Calorimetry (DSC) was used to assess the thermal stability of Ab1 Fc variants. A summary which includes the mid-point of denaturation (Tm) for the transitions is shown in Table 2. HuIgG1 (WT), huIgG1 AA and huIgG2 have similar Tm1 (C_H_2 domain) transitions, but huIgG1 FES, huIgG1σ, huIgG2σ, huIgG4 PAA, huIgG4σ1 and huIgG4σ2 show lower Tm1 transition values, suggesting reduced stability of its C_H_2 domain attributable to the presence of mutations (Table 1). Ab1 huIgG2 Tm1 transition is 71.6 ± 0.1 °C, while the huIgG2σ variant is 62.0 ± 0.2 °C for Tm1. The Ab1 huIgG4 PAA shows a first transition at 69.5 ± 0.1 °C, while the huIgG4σ1 is at 62.0 ± 0.2 °C and the huIgG4σ2 is at 61.2 ± 0.6 °C, suggesting that the huIgG4 PAA is more thermally stable than its variants. The huIgG4 PAA differences are interesting given that the only sequence differences are in the first two or three residues in the lower hinge (Table 1). The Tm2 and Tm3 indicating the Fab and C_H_3 transitions are included for comparison, but generally show normal antibody transition profiles between 69 and 83 °C [46].

Colloidal stability was evaluated over a temperature range by static light scattering (SLS) at two different wavelengths (Table 2). The scattering intensity at 266 nm is more sensitive to fluctuations of smaller aggregates. Measurements of scattering intensity at 473 nm are useful for detection of larger aggregates. The SLS data indicate the onset of aggregation (Tagg) for each antibody sample. Results comparing the antibody variants show that huIgG1 variants have higher aggregation onset temperatures around 68 °C (SLS at 266 nm) compared to the huIgG2 or huIgG4 variants (Tagg = 64–65 °C using SLS at 266 nm). SLS measurements at 473 nm display a similar profile.

#### 2.8.2. Stability of Concentrated Samples

Samples were concentrated from their initial concentration of 1–2 mg/mL to 40–50 mg/mL and stored at 4 °C. Table 3 summarizes the antibody concentrations and Table 4 summarizes the size exclusion chromatography (SEC) analyses at time of lot release, week 1, week 2, week 3, and week 4 post-release. All molecules show stable antibody concentrations for up to 4 weeks storage at 2–8 °C in PBS. The increasing concentration values observed for all samples with time are attributed to sample evaporation during storage. Aliquots at these time points were diluted and analyzed by SEC. All samples appear visibly clear and SEC analyses results indicate that all molecules at high concentrations consist of greater than 95% monodisperse monomeric antibody.

To obtain a more complete picture of the stability of the Fc variants, protein melting points and aggregation onset temperature were measured for each by a combination of intrinsic fluorescence and static light scattering. Table 5 summarizes the mid-point values for thermal transition, Tm1, and for onset of aggregation at Tagg = 266 nm. Tm values for the concentrated antibody variants are similar between the release lot and the week 4 samples. Tagg values of the 266 nm values are also similar for the release lot and week 4 samples. Thus, the variants have not changed their conformational or colloidal stabilities at high concentrations.

#### 2.8.3. Stability of Low pH Treated Antibody Fc Variants

To assess whether the Fc variants are stable after low pH treatment for viral load reduction in manufacturing, antibody samples were treated at pH 3.5 for 6 h and dialyzed back to pH 7.4 in PBS. Dynamic light scattering analyses were performed to quantitate presence of soluble aggregates and sub-visible particles. Table 6 shows the average hydrodynamic radius (R_h_), percent polydispersity (% Pd) values, and percent mass comparison between the release lot and the low pH treated samples. All samples yielded a peak with an average radius of 5–7 nm, average % Pd of less than 15%, and an average % Mass of greater than 99%. These features are indicative of a mono-dispersed species for the antibody variants.

### 2.9. Fc Crystal Structures

To assess the impact of the silencing mutations on the structure of the Fc, crystal structures of the Fc fragments of huIgG1σ, huIgG4σ1, and huIgG4σ2 were determined to resolutions of 1.90 Å, 1.90 Å, and 1.85 Å, respectively. Data collection and refinement statistics are summarized in Table 7. Crystals of all three Fc constructs belonged to space group P2_1_2_1_2_1_, and had similar unit cell parameters. This isomorphism reflects a similarity in Fc packing within the crystal lattice where one intact Fc dimer is present in the asymmetric unit. In this crystal form, the A chain C_H_2 domain is less ordered as evidenced by the reduced quality of the electron density map and elevated temperature factors of the atoms. The reason for this is that the C_H_2 domains of the A and B chains have a difference in the crystal contacts. For the three Fc structures reported here and excluding the glycan contributions, the B chain has an average of 2082 ± 186 Å^2^ of buried surface area through interaction with symmetry related Fc molecules compared with only 1477 ± 181 Å^2^ of buried surface area for chain A. Consequently, for the three structures, the average B-factor calculated over residues 238–339 and glycan residues for chains B and A was 37.6 ± 4.5 Å^2^ and 54.1 ± 1.8 Å^2^, respectively.

**Figure 12:**
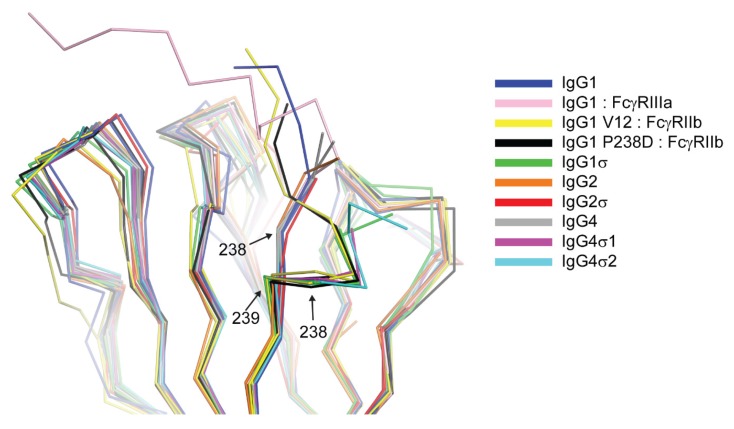
Structural consequence of mutation at position 238 reveals an altered conformation of the lower hinge. Backbone alignment of Fc C_H_2 domains of huIgG1 (Protein Data Bank (PDB) 3AVE [40]), huIgG1 in complex with FcγRIIIa (chain B, PDB 3SGJ [47]), huIgG1 V12 variant in complex with FcγRIIb (PDB 3WJL [48]), huIgG1 P238D in complex with FcγRIIb (PDB 3WJJ [48]), huIgG1σ, huIgG2 (PDB 4HAF [49]), huIgG2σ (PDB 4L4J [29]), huIgG4 (PDB 4C54 [50]), huIgG4σ1, huIgG4σ2. The Cα positions of residues 238 and 239 are labeled.

The huIgG1σ Fc structure includes residues G236-S444 and A235-S444 for chains A and B, respectively, which are only those observed in the electron density map (Figure S1). The structure reveals conformational differences in ordered residues N-terminal to V240 and within the BC loop (residues S267 to V273) relative to wild-type huIgG1 Fc (e.g., PDB 3AVE [40]) and receptor-bound Fc structures (e.g., PDB 3SGK [47]). These regions can directly influence interactions with FcγRs. The lower hinge regions of wild-type huIgG1 structures, including those in complex with FcγRs, show extended conformations while that of huIgG1σ has a kinked conformation at S239 (Figure 12 and Figure S1). The backbone psi angle value for S239 averaged over both chains in the huIgG1σ Fc structure is 10° compared to an average value of 145° for wild-type huIgG1 in structure 3AVE [40]. It should be noted that the altered lower hinge conformation is not a requirement of crystal packing (Figure S2). Interestingly, the kinked conformation is observed in structures of two other engineered Fcs in complex with FcγRIIb, a P238D mutant (PDB 3WJJ [48]) and an Fc having the P238D mutation in addition to six other mutations (PDB 3WJL [48]) (Figure 12).

The structure of huIgG4σ1 Fc includes residues A237-L443 and G236-S444 for chains A and B, respectively, that were modeled into the visible electron density. For the huIgG4σ2 Fc structure, residues S238-L443 and E233-S444 were modeled for the respective chains (Figure S1). The lower hinge residues in both chains of the huIgG4σ1 Fc structure and in chain B of the huIgG4σ2 Fc structure adopt a kinked conformation like that described above for huIgG1σ (Figure 12 and Figure S1). In contrast, the same residues in chain A of the huIgG4σ2 structure adopt a wild-type-like extended conformation (Figure 12). All these Fcs having a kinked lower hinge also contain the mutation of P238, however, the structure of huIgG2σ (PDB 4L4J [29]) and chain A of huIgG4σ2 indicate that this mutation does not restrict the conformation to the kinked form.

**Figure 13:**
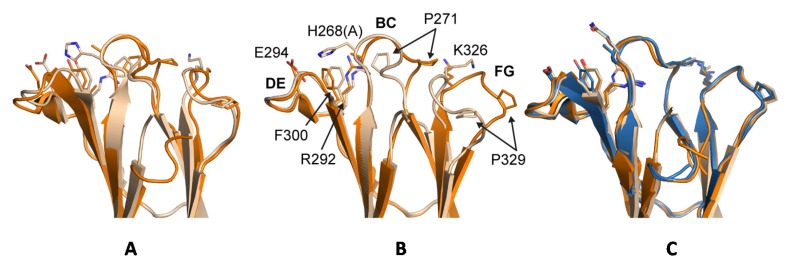
Conformational differences in BC and FG loops in WT and σ variants of huIgG. (**A**) Overlay of Ch2 domains (cartoon) from huIgG1 (wheat, PDB 3AVE chain A [40]) and huIgG1σ (orange, chain B); (**B**) huIgG2 (wheat, PDB 4HAF chain A [49]) and huIgG2σ (orange, PDB 4L4J chain A [29]); (**C**) huIgG4 (wheat, PDB 4C54 chain A [50]), huIgG4 σ1 (blue, chains A and B), and huIgG4 σ2 (orange, chains A and B). BC and FG loops are in the flipped-in conformation in huIgG1 (middle, wheat) and flipped-out conformation in huIgG2σ (middle, orange). Residues at positions 268, 271, 292, 294, 300, 326, and 329 are shown as sticks and are labeled in the middle panel with huIgG2 sequence (mutation at position 268 in huIgG2σ given in parentheses). N-terminal residues 234–236 are omitted from the cartoon of huIgG1 for clarity, and the alternate conformations modeled for the BC loop of huIgG1σ are shown.

Previous crystal structures of huIgG1, huIgG2 and huIgG4 have shown two distinct conformations for the BC loop (residues 267 to 273) and the FG loop (residues 322 to 332) that seem to correlate with the ability to bind to FcγRs. Denoting the two conformations as ‘flipped-in’ and ‘flipped-out’, both loops are in the flipped-in conformation in structures of wild-type huIgG1 Fc (PDB 3AVE [40]) and huIgG2 Fc (PDB 4HAF [49]), whereas they adopt the flipped-out conformation in structures of huIgG2σ (PDB 4L4J [29]), huIgG4 wild-type (PDB 4C54 [50]) and in huIgG4σ1 and huIgG4σ2 (Figure 13). Interestingly, the electron density map for huIgG1σ Fc reveals a deviation from this pattern such that the primary conformation of the BC loop is the flipped-out conformation while that of the FG loop is the wild-type flipped-in conformation. In chain A, for which the density is poorer, these are the only conformations evident. In chain B, density is also present for the flipped-in conformation of the BC loop, and this conformation refined to an occupancy of 40%. Density for the FG loop in chain B suggests an increased mobility for this loop, but was not well enough defined to model a second conformation. Whereas crystal packing precludes a flipped-out FG conformation for one of the two Fc chains in the huIgG1σ structure, a flipped-out conformation could be tolerated for the second (Figure S3). In the wild-type huIgG1 Fc structure (PDB 3AVE [40]), residue P271 in a flipped-out conformation of the BC loop would clash with the flipped-in FG loop. However, this clash is avoided in huIgG1σ by a deflection of the FG loop as a rigid body (Figure 13). In addition to the deviation in conformations of the BC and FG loops for the huIgG1σ Fc, the structural studies suggest an increased mobility for these two loops that may play a direct role in how this Fc interacts with the FcγRs.

### 2.10. Molecular Dynamics Simulations of the Fc FG and BC Loops

**Figure 14:**
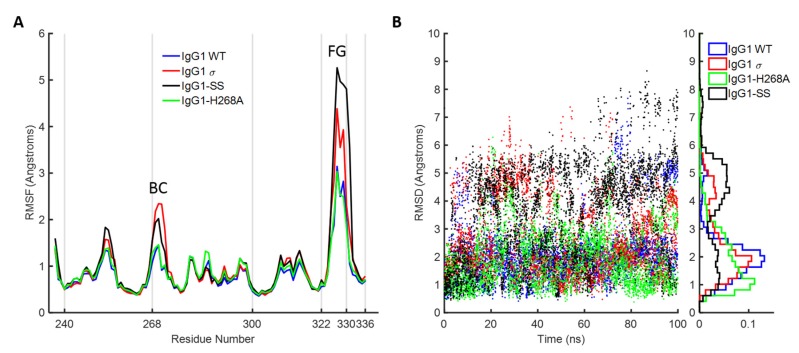
Molecular dynamics simulation of huIgG1 wild-type and mutants. (**A**) Root mean squared fluctuations (RMSF) of Cα atoms of chain B of C_H_2 domain. The trajectories are aligned on the Cα atoms with respect to the non-BC and non-FG loop residues of chain B of C_H_2 domain of the corresponding crystal structures. The peaks corresponding to the BC and FG loop are marked. The two loops are more flexible in huIgG1σ (red) as compared to wild-type (blue). The fluctuations in huIgG1-H268A (green, containing only the BC loop mutation) are closer to wild-type while those in huIgG1-SS (A330S P331S) (black, contains only the FG loop mutations) are closer to the silent variant. (**B**) Root mean squared deviation (RMSD) of the Cα atoms of the FG loop with respect to the flipped-in conformation. RMSD values are shown for all frames of the 100-ns trajectory. The right panel shows a normalized histogram of the RMSD values.

**Figure 15:**
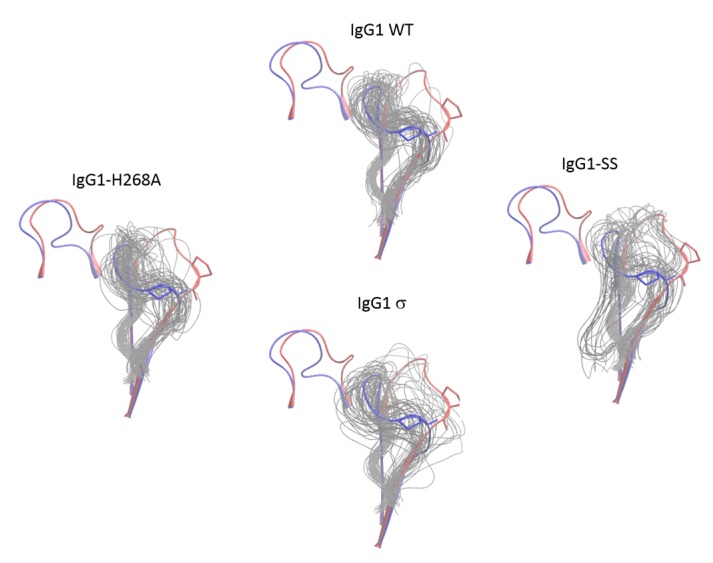
Conformational distribution of the FG loop of chain B of C_H_2 domain from molecular dynamics simulations of huIgG1 wild-type and mutants. Each panel shows the flipped-in (blue cartoon) and flipped-out (red cartoon) conformations of the two loops. The flipped-in conformation is from the huIgG1 WT (PDB 3AVE [40]) while the flipped-out conformation is from huIgG4 WT (PDB 4C54 [50]). All simulations were started from the flipped-in conformation of the FG loop. The panels show fifty conformations (gray) sampled at an interval of 2 ns after aligning the molecular dynamics (MD) trajectory on the Cα atoms of the non-BC and non-FG residues of the C_H_2 domain of chain B in the corresponding crystal structure. Figure S4 shows the conformation of P329 in the above loop conformations. The FG loop of huIgG1σ and huIgG1-SS is significantly more flexible than that of huIgG1 WT and huIgG1-H268A.

To further investigate the dynamic stability of the loops in wild-type huIgG1 and huIgG1σ that could potentially influence FcγR binding, explicit solvent molecular dynamics simulations were performed starting from the crystal structures of wild-type huIgG1 (PDB 3AVE [40]) and huIgG1σ. Both simulations were initiated from structures in which the FG loop was in the flipped-in conformation. For the wild-type simulation, the FG loop remained close (within 2Å C_α_ RMSD) to the flipped-in conformation for 57.1% of the simulation as compared to 44.4% in the case of huIgG1σ where the loop was found to be substantially more flexible (Figure 14 and Figure 15). Notably, the flipped-out conformational state of the FG loop was not found to have significant population in either simulation. Figure S4 shows the spatial distribution of P329 in the FG loop, a key residue for binding to various FcγRs (e.g., [29,51]). The distribution of P329 is more tightly clustered in wild-type than in huIgG1σ suggesting that the FG loop in wild-type is preconfigured for receptor binding.

**Figure 16:**
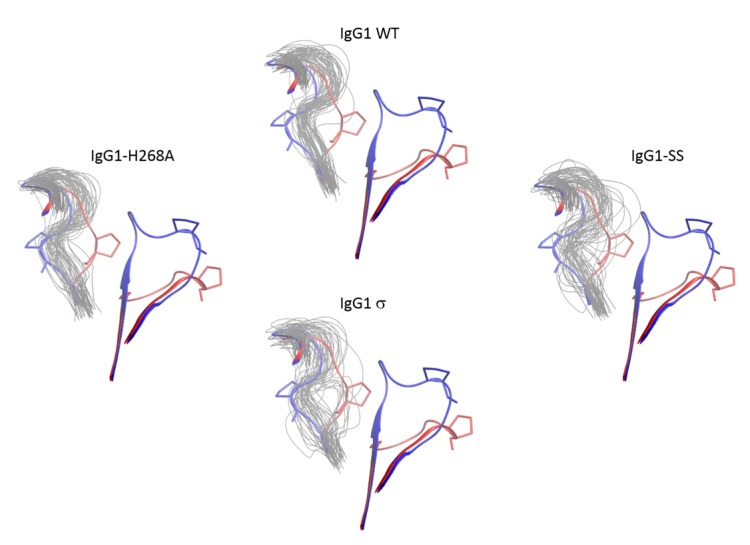
Conformational distribution of the BC loop of chain B of C_H_2 domain from molecular dynamics simulations of huIgG1 wild-type and mutants. Each panel shows the flipped-in (blue cartoon) and flipped-out (red cartoon) conformations of the two loops. The flipped-in conformation is from the huIgG1 WT (PDB 3AVE [40]) while the flipped-out conformation is from huIgG4 WT (PDB 4C54 [50]). The huIgG1σ was started with the BC loop in the flipped-out conformation while the other simulations started from the flipped-in conformation. The panels show fifty conformations (gray) sampled at an interval of 2 ns after aligning the MD trajectory on the Cα atoms of the non-BC and non-FG residues of the C_H_2 domain of chain B in the corresponding crystal structure. Figure S5 shows the conformation of P271 in the above loop conformations. In the wild-type and huIgG1-H268A simulations, the BC loop remained close to the initial flipped-in conformation of the loop. The huIgG1σ showed a broader distribution around the flipped-in state while huIgG1σ samples both flipped-in and flipped-out states though the flipped-in state was dominant.

In addition to the FG loop, the simulations show that the BC loop is also more flexible in huIgG1σ than in the wild-type huIgG1 (Figure 14 and Figure 16). In the wild-type simulation, the BC loop stays close to the initial flipped-in conformation throughout the simulation, but occupied both flipped-in and flipped-out states in the huIgG1σ simulation (Figure 16). This is clearly illustrated by the spatial distribution of residue P271 in the BC loop which shows a single cluster for wild-type but two distinct clusters for huIgG1σ (Figure S5) corresponding to the two conformational states. Interestingly, although the huIgG1σ simulation was initiated from the flipped-out conformation of the BC loop, the flipped-in conformation turns out to be more populated. The simulations may not be long enough to converge the populations of the two states, but strongly suggest that the BC loop of silent huIgG1σ is more flexible than that of the wild-type.

HuIgG1 and huIgG1σ differ at position 268 (histidine in huIgG1, alanine in huIgG1σ) in the BC loop, and at positions 330 (alanine in huIgG1, serine in huIgG1σ) and 331 (proline in huIgG1, serine in huIgG1σ) in the FG loop. To investigate the impact of these mutations on the flexibility of the two loops, two additional simulations were performed after modelling the mutations in the huIgG1 wild-type crystal structure. One simulation (referred to as huIgG1-H268A) contained only the BC loop mutation, and the other (referred to as huIgG1-SS) contained only the two FG loop mutations. The flexibility of both loops in the huIgG1-H268A simulation was nearly identical to that in the wild-type simulation while the flexibility in huIgG1-SS simulation was closer to that in the huIgG1σ simulation (Figure 14, Figure 15, Figure S5 and Figure S6). The mutant simulations suggest that the A330S P331S mutations in the FG loop are primarily responsible for the increased flexibility of the two loops in huIgG1σ; the H268A mutation in the BC loop has minimal impact.

## 3. Discussion

Many therapeutic antibodies and Fc fusion proteins employ Fc activity as part of their mechanisms of action. Fc engagement with FcγR can activate myeloid cell and NK cell activity as well as the generation of reactive species that induce apoptosis and release of inflammatory cytokines which are important for eliminating unwanted target cells (i.e., tumor cells) [52,53,54,55]. However, antibody targeting to cell surface receptors can pose potential safety risks since Fc activity could elicit ADCC, ADCP, CDC, and/or apoptosis which can cause tissue damage, depletion of target cells, and infusion reactions. Although numerous antibody engineering efforts to silence the Fc activity of huIgG1 and huIgG2 have been reported [3,16,24,25,33,34,56], we describe alternative novel mutations in huIgG1σ, huIgG4σ1, huIgG4σ2, which can provide design choices for future Fc silent huIgG antibodies or Fc fusions.

Several antibody panels (Ab1, Ab2, Ab3, Ab4, Ab5) with Fc silent mutations were constructed, purified, and tested in vitro for Fc binding and function. The huIgG1σ, huIgG4σ1, and huIgG4σ2 antibodies tested as immune complexes had minimal binding to FcγRI, FcγRIIa, FcγRIIb, and FcγRIIIa, compared to huIgG1 WT using concentration ranges that could be found in clinical dosing. Since the TNFα on target cells and FcγRIII on effector cells are both multivalent, the engagement of IgG1 molecules to both cells would involve avidity. Thus, ADCC activity and the induction of immune effector function which depend on avidity and could occur at lower concentrations compared with monovalent antigen (i.e., in plate assays) [57]. Therefore, the aforementioned cell based assays provide a more sensitive measure of the degree of silencing effector function and provide a meaningful biological readout. HuIgG1σ, huIgG4σ1 and huIgG4σ2 were more silent in ADCC activity when compared to huIgG1 AA, IgG1 FES, and IgG4 PAA. HuIgG4 WT was not included in the comparisons because such molecules can exchange to half-molecules in a dynamic process (Fab-arm exchange) [35]. Instead, subsequent studies were compared with HuIgG4 PAA, which has a stabilized hinge, reduced effector function, and has been used in therapeutic antibodies [25].

In vivo studies emphasize again the lack of immune functionality with the huIgG sigma variants. Results using FcγR-hu mice demonstrated significantly lower levels of T cell dependent activation (CD69 and CD25 upregulation) with the sigma variants compared to huIgG1 AA, IgG1 FES, and huIgG4 PAA. In vivo half-life in a transgenic mouse model of human FcRn and in cynomolgus monkeys indicated that the silent mutations did not alter PK properties compared to normal huIgG. In addition, potential immunogenicity, evaluated in silico (for protein sequence “hot spots” that favor immune response initiation) predicts minimal immunogenic risk for these silent Fc variants. Although ex vivo immune responses measured by T cell proliferation and IL-2 secretion, were not tested here, the huIgG2σ (reported previously) shows relatively low risk of clinical immunogenicity as determined by comparing the frequency and magnitude of ex vivo T cell responses [29]. The IgG1 and IgG4 sigma variants with very similar mutations and low predicted immunogenic risk are likely to have similar non-immunogenic profiles.

The impact of huIgG1σ, huIgG4σ1 and huIgG4σ2 mutations on biophysical properties was assessed for their effect on manufacturing. Thermal stress for all the sigma variants compared to their wild-type versions, suggests lower thermal stabilities in the C_H_2 and hinge regions. However, there is little evidence that reduced thermal stability of these magnitudes translates into developability issues or in vivo stability issues. All Fc variants are stable for 4 weeks when concentrated (to 40–50 mg/ML), or subjected to low pH stress. Also, there are no additional post-translational modifications or significant changes in solution particle size associated with these Fc mutations. 

In the analysis of Fc:FcγR structures, the packing of P329 in the C_H_2 domain FG loop between two tryptophan side chains in the Fc receptor is a conserved feature of huIgG Fc interaction with FcγRI (e.g., 4W4O [51]), FcγRIIb (e.g., 3WJJ [48]), and FcγRIIIa (e.g., 3SGJ [47]). The receptor bound conformation of the FG loop is like its conformation in apo-structures of huIgG1 Fc and huIgG2 Fc suggesting that in these subtypes, the FG loop is preconfigured for receptor engagement (Figure S6). The structure of huIgG2σ, an engineered silent variant of huIgG2, first revealed a unique flipped conformation for the FG loop that was proposed to be responsible in part for the diminished receptor interaction of this variant. The same structure also revealed a unique, flipped conformation of the BC loop relative to wild-type structures of huIgG1 Fc and huIgG2 Fc. Herein, we denote the conformations of the BC and FG loops as observed in a prototypical structure of huIgG1 wild-type Fc (e.g., PDB 3AVE [40]) as flipped-in and the altered conformations of the same loops observed in the crystal structure of huIgG2σ (PDB 4L4J [29]) as flipped-out (Figure 13).

It has been proposed that, in the case of huIgG2σ, the H268A mutation in the BC loop abolished an electrostatic interaction with E294 in the DE loop resulting in the observed flipped conformation of the BC loop which in turn triggered the flip of the FG loop [29]. Indeed, alignment of the C_H_2 domains from crystal structures of huIgG2 Fc and huIgG2σ Fc suggested that residue P271 in a flipped-out BC loop could clash with K326 in a flipped-in FG loop (Figure 4). A similar flipped-out conformation of the FG loop has since been observed in crystal structures involving huIgG4 Fc [50] (Figure 4). Davies et al. have suggested that sequence differences within the FG loop between huIgG1 and huIgG4 were primarily responsible for the FG loop flip in the latter, and not the absence of H268 [50]. Consistently, one C_H_2 domain in PDB 4D2N [58], a structure of deglycosylated huIgG4 Fc, reveals an FG loop that, although partially disordered, appears to have a flipped-out conformation while the BC loop is maintained in a flipped-in conformation. The present crystal structure of the huIgG1σ Fc demonstrated the possibility of the coexistence of a flipped-out BC loop and a flipped-in FG loop. A clash between P271 and K326 was avoided by a rigid body displacement rather than conformational flip of the FG loop away from the flipped BC loop (Figure 4). Furthermore, MD simulations of a single C_H_2 domain of huIgG1 wild-type and mutants were consistent with the hypothesis that positions 330 and 331 are of primary importance to the conformational stability of the FG loop. The simulations showed that the SS (A330S P331S) mutations dramatically increase the flexibility of the FG loop even in the absence of the H268A mutation. Also, just the H268A mutation, in the absence of the FG loop mutations, only marginally increases the flexibility of the two loops relative to wild-type.

The results of our in vitro and in vivo studies demonstrated that huIgG4σ1 and huIgG4σ2 are more silent than huIgG4 PAA as discussed above. Given that all the mutations for these variants were localized to the lower hinge region (Figure 2) and that structurally the dispositions of the BC and FG loops were identical to those observed in structures of wild-type huIgG4 Fc (Figure 4), these mutations could function either by directly disrupting hinge:receptor interactions or indirectly by altering lower hinge backbone conformation. Structures of huIgG1 in complex with FcγRI and FcγRIIIa have shown the importance for L235 in receptor engagement (Figure S6). This residue was mutated to alanine in both huIgG1σ as well as huIgG4σ1/2, and this mutation likely has a direct effect on receptor engagement. In contrast, an altered conformation of ordered lower hinge residues N-terminal to position 240 observed in structures of huIgG1σ, huIgG4σ1, and huIgG4σ2 relative to huIgG1 wild-type was like that observed in structures of huIgG1 P238D and huIgG1 C220S, E233D, G237D, P238D, H268D, P271G, A330R in complex with FcγRIIb (Figure 3). All Fc variants demonstrating this kinked conformation commonly share mutation of a conserved proline at position 238 and have an altered ability to engage Fc receptor. Thus, although P238 does make contacts with Fc receptor, it is likely that this residue also plays an important indirect role in maintaining receptor affinity, biasing the structure of the lower hinge toward a conformation competent for receptor engagement.

In summary, antibodies with huIgG1σ, huIgG4σ1, and huIgG4σ2 Fc regions have demonstrated minimal FcγR interactions by in vitro and in vivo methods. Immunogenicity, developability, and PK risks of these variants have been evaluated and determined to be comparable to that the huIgG1 WT (Table 8) and huIgG4 PAA. Thus, we propose that the huIgG1σ, huIgG4σ1 and huIgG4σ2 variants, which offer several IgG subtype choices, be considered along with the existing silent Fc structures for incorporation into antibody based biotherapeutic molecules.

## 4. Materials and Methods

### 4.1. Antibodies

Ab1 is a huIgG1 kappa antibody specific for tumor necrosis factor alpha (TNFα); Ab2 is a huIgG1 kappa monoclonal antibody specific for F glycoprotein of Respiratory Syncytial Virus (RSV); Ab3 is a huIgG1 bispecific antibody targeted against RSV and glycoprotein gp120 of the human immunodeficiency virus (HIV) envelope; Ab4 is an anti-mouse CD3ε-chain Ab, 145-2C11, and Ab5 is TA99, an anti-gp75 antibody which targets gp75 antigen on B16F10 melanoma cells [45,59]. H435A is a mutation which reduces binding to FcRn [60]. LoF refers to low fucosylated IgG which has increased ADCC/ADCP effector function in vitro and in vivo [42]. R10Z8E9 is a mouse anti-huIgG antibody that is specific for the C_H_2 domain [61]. All antibodies were produced and purified at Sino Biologics by transient HEK cell transfection. Antibodies were purified using standard protein A chromatography and confirmed to be greater than 95% purity and low in endotoxin prior to experiments. The bispecific antibody was made using the DuoBody^®^ technology (Genmab, Copenhagen, Denmark) [62], and confirmed to be greater than 95% purity.

### 4.2. Cell Lines

K2 cells are Sp2/0 mouse myeloma cells which express a mutant, transmembrane form of human TNFα [41,63]. K2 cells were cultured at 37 °C, 5% CO_2_, in Iscove’s Modified Dulbecco’s Medium (IMDM) with GlutaMAX and 5% (*v*/*v*) heat-inactivated fetal bovine serum (FBS), 1× non-essential amino acids (NEAA), 1× sodium pyruvate, 0.5 μg/mL mycophenolic acid, 2.5 μg/mL hypoxanthine, and 50 μg/mL xanthine (MHX). Media components were purchased from Life Technologies (as 100×, Carlsbad, CA, USA) and the MHX components from Sigma (St. Louis, MO, USA).

B16F10 mouse melanoma cell line was obtained from the American Tissue Culture Collection. Cells were cultured in RPMI 1640 supplemented with 10% (*v*/*v*) FBS, 1× NEAA, and 1× sodium pyruvate.

### 4.3. Fc Gamma Receptor (FcγR) Binding

Binding of Ab1 variants to human FcγRs was assessed using an AlphaScreen (PerkinElmer, Boston, MA, USA) bead assay in a competition binding format. FcγRs were purchased from R&D Systems or Sino Biological Inc. (Beijing, China). Since the outcome for huIgG with a silent Fc in a FcγR binding assay may be a negative result, assay sensitivity was increased by introducing avidity to the test samples via cross-linking. This was achieved using a goat F(ab′)_2_ anti-huIgG F(ab′)_2_-specific fragment (Jackson ImmunoResearch, West Grove, PA, USA) in 1:1 molar ratio with the test huIgGs. Cross-linked test antibodies (Thermo Fisher Scientific, Waltham, USA) were added to Corning white half-well 96-well assay plates (Corning Inc., Corning, NY, USA) at the designated concentrations in competition with biotin-labeled huIgG Fc fragment (biot-Fc) at either 1 μg/mL (for FcγRI, -RIIa, and -RIIb assays) or 5 μg/mL (for FcγRIIIa assays). Biotinylated Fc fragment was used to prevent binding to the biot-Fc by the test article cross-linker described above. FcγRs as specified were added to a 200 ng/mL final concentration. Nickel chelate acceptor beads were added; followed by streptavidin donor beads. Plates were covered with foil adhesive plate sealers to protect from light, and placed on an orbital plate shaker with gentle shaking for 45 min at room temperature (RT). Subsequently, plates were read on the EnVision multi-label plate reader (Perkin-Elmer), and data plotted with GraphPad Prism v6.0 software (GraphPad, San Diego, CA, USA).

### 4.4. Competitive Binding to Recombinant Human FcRn

A competitive binding assay was used to assess relative affinities of different antibody samples to recombinant human FcRn (in-house expressed with transmembrane and cytoplasmic domains of FcRn replaced with a poly-histidine affinity tag). Ninety-six-well copper-coated plates (Thermo Scientific) were used to capture FcRn-His6 at 4 μg/mL in PBS, after which plates were washed with 0.15 M NaCl, 0.02% (*w*/*v*) Tween 20, and then incubated with blocking reagent (0.05 M MES, 0.025% (*w*/*v*) bovine serum albumin, 0.001% (*w*/*v*) Tween-20, pH 6.0, 10% (*v*/*v*) ChemiBlocker from Sigma-Aldrich, St. Louis, MO, USA. Plates were washed and serial dilutions of competitor test antibody in blocking reagent were added to plates in the presence of a fixed 4 μg/mL concentration of an indicator antibody (a biotinylated huIgG1). Plates were incubated at RT for 1 h, washed 3 times, and then incubated with a 1:10,000 dilution of HRP (Jackson ImmunoResearch Laboratories) at room temperature (RT) for 30 min to bind biotinylated antibody. Plates were washed and bound streptavidin-HRP was detected by adding TMB peroxidase substrate (Fitzgerald, Acton, MA, USA). Color development was stopped by addition of 0.5 M HCl. Optical densities were determined with a SpectraMax Plus384 plate reader (Molecular Devices, Sunnyvale, CA, USA) at 450 nm wavelength, and data plotted with GraphPad Prism v6.0 software.

### 4.5. Antibody-Dependent Cell-Mediated Cytotoxicity (ADCC)

Peripheral mononuclear cells (PBMCs) were isolated from heparinized blood from in-house donors. Eighty (80) mL of blood from 2 donors were typically used per experiment. Blood was diluted 2-fold with PBS and 30 mL was layered over 15 mL of Ficoll-Paque (Perkin-Elmer, Waltham, MA, USA) in a 50 mL conical centrifuge tube. Tubes were centrifuged at 400× *g* at RT for 30 min. The upper plasma supernatant was removed and the interface white cell layer was collected and washed twice with PBS to remove Ficoll and majority of platelets. Cells were resuspended in IMDM-5% heat inactivated FBS with 1× sodium pyruvate, 1× NEAA, and 1× penicillin-streptomycin (100× from Life Technologies, Carlsbad, CA, USA) for culturing overnight at 37 °C, 5% CO_2_.

K2 cells were used as target cells at a ratio of 50 effector cells per 1 target cell. Target cells were pre-labeled with BATDA (bis (acetoxymethyl) 2,2′:6′,2′′-terpyridine-6,′′-dicarboxylate, DELFIA^®^ EuTDA, PerkinElmer) for 25 min at 37 °C, washed 3 times in culture medium (IMDM with Glutamax, 10% (*v*/*v*) heat-inactivated Fetal Bovine serum (FBS), 1× non-essential amino acids (NEAA), 1× sodium pyruvate, 1×, penicillin-streptomycin; all from Life Technologies and resuspended in culture medium. Target cells (2 × 10^5^ cells/mL, 50 μL) were added to test antibody (100 μL) in 96-well U-bottom plates, then effector cells (1 × 10^7^ cells/mL, 50 μL) were added. Plates were centrifuged at 200× *g* for 3 min, incubated at 37 °C for 2 h, and then centrifuged again at 200× *g* for 3 min. A total of 20 μL of supernatant was removed per well, and cell lysis was measured by the addition of 200 μL of the DELFIA Europium-based reagent (PerkinElmer). Fluorescence was measured using an Envision 2101 Reader (PerkinElmer). Data were normalized to maximal cytotoxicity with 0.7% (*w*/*v*) Triton X-100 (Sigma-Aldrich, St. Louis, MO, USA) or 10% (*v*/*v*) Lysis Buffer (DELFIA^®^ PerkinElmer) and minimal lysis using target cells in the absence of any Ab. Samples were tested in duplicate. Percent specific lysis was calculated to be (sample lysis − minimal lysis) divided by (maximal lysis-minimal lysis) × 100. Data were fit to a sigmoidal dose-response model using GraphPad Prism v6.0 software.

### 4.6. Antibody-Dependent Cellular Phagocytosis (ADCP)

Monocytes were isolated from human PBMCs using a Monocyte Isolation Kit (Miltenyi, Auburn, AL, USA) and differentiated into macrophages for 1 week by culturing with 10 ng/mL recombinant human granulocyte-macrophage colony-stimulating factor (GM-CSF) and 10 ng/mL recombinant human IL-4 (both from R & D Systems, Minneapolis, MN, USA) in IMDM with Glutamax, 10% heat-inactivated FBS, 1× NEAA, 1× sodium pyruvate, 1× penicillin-streptomycin. Macrophages were labeled with PKH26 (fluorescent dye for cell membrane, Sigma) and K2 cells were labeled with PKH67 (Sigma). Labeled cells in IMDM-10% (*v*/*v*) heat-inactivated FBS media without phenol red were incubated for 5 h with a macrophage to K2 cell ratio of 1 effector to 1 target cell in the presence of test antibody. Two-color flow cytometry analyses were performed with a MACSQuant Flow Cytometer (Miltenyi) using optimal compensation in the B1 (PKH67) and B2 (PKH26) channels and gating on single cells. Dual-labeled cells (PKH26+/PKH67+) were considered to represent phagocytosis of K2 target cells by macrophages. Percent ADCP or phagocytosis of target cells was calculated to be 100× number of dual-labeled cells (macrophage + target) divided by the total number of target cells in the population (phagocytosed + non-phagocytosed) after >50,000 cell counts. The percent specific ADCP was obtained by subtracting from each sample the background value (macrophage + target incubated without Ab) [5].

### 4.7. Complement-Dependent Cytotoxicity (CDC)

K2 cells were used as target cells for CDC assays. A total of 50 μL of cells was added to wells of a 96-well plates for a final concentration of 8 × 10^4^ cells per well in IMDM with Glutamax, 10% (*w*/*v*) heat-inactivated PBS, 1× NEAA, 1× sodium pyruvate, 1x penicillin-streptomycin. An additional 50 μL was added to the wells with or without test Abs and plates were incubated at 37 °C for 2 h. A total of 50 μL of 10% (*w*/*v*) rabbit complement (Invitrogen, Carlsbad, CA, USA) was added to the wells, and plates were incubated for 20 min at 37 °C. All samples were performed in triplicate. The plates were centrifuged at 200× *g* for 3 min, 50 μL of supernatant was removed to separate plates, and CDC was measured with a LDH cytotoxicity detection kit (Roche, Indianapolis, IN, USA). Absorbance was measured using a Spectra Max Plus 384 (PerkinElmer). Data were fit to a sigmoidal dose-response model using GraphPad Prism v6.0 software. Maximal cytotoxicity was obtained with Triton X-100 (Sigma-Aldrich) and spontaneous release with cells and complement alone. Specific cell lysis was calculated as follows: Cytotoxicity (%) = 100 × (optical density (OD) of sample − OD of spontaneous release)/(OD of maximal lysis − OD of spontaneous release).

### 4.8. Animals

The FcγR-humanized (FcγR-hu) mice used in the T-cell activations studies express the different human FcγRs: CD16a (FcγRIIIa), CD16b (FcγRIIIb), CD32a (FcγRIIa), CD32b (FcγRIIb) and CD64 (FcγR1) and their endogenous mouse FcγRs have been inactivated [43]. Three strains of these C57BL/6 mice (8–10 weeks old) were used: FcγRα null females, FcγR-hu hemizygous (hemi) females, and FcγR-hu homozygous (homo) females.

Human FcRn transgenic animals (8–10 weeks old) used in PK studies were derived from C57BL/6 mice [64]. Tg32 mice (B6.Cg-Fcgrt^tmLDcr^Tg(FCGRT)32Dcr from The Jackson Laboratory) have their endogenous mouse FcRn α gene knocked out and are transgenic with the human FcRn α gene under the control of the native human gene promoter [65,66]. The FcRn transgenic strain show clinical chemical parameters like those found in wild-type mice except for endogenous huIgG levels, which are greatly reduced in these mice [67]. Tg32 hemi referred to mice hemizygous for the FcRn transgene, the latter derived by mating homozygous transgenic mice with FcRn α knockout mice (transgene copy number reduced by half).

Naive cynomolgus monkeys used in the PK study at WuXi AppTec., (Suzhong, China) were approximately 2 to 3.5 years old and weighed between 2.5 and 3.0 kg.

### 4.9. T Cell Activation

FcγR-hu mice were used in the in vivo binding and T cell activation studies. The preliminary study was done with three strains of mice (8–10 weeks old): FcRα null females, FcγR-hu (hemi) females, and FcγR-hu homozygous (homo) females. Test antibody was injected into the intraperitoneal cavity of the mice at 0.5 mg/kg, 10 mL/kg. Approximately 24 h later, mice were euthanized by CO_2_ asphyxiation and their spleens removed and placed into tubes containing cold RPMI-1640, 5% (*v*/*v*) heat-inactivated FBS, 1% (*w*/*v*) L-glutamine.

Mouse splenocytes were prepared from 3 to 6 mice as single-cell suspensions from each individual spleen on the day of harvest. They were washed with media, followed by anucleated red blood cell depletion using hypotonic RBC lysis solution (eBiosciences). Splenocytes were analyzed by flow cytometry for cell surface expression of CD25 and CD69 T cell activation markers. Cells were resuspended in staining buffer consisting of PBS for viability staining (IR Live Dead, Invitrogen), washed, and then incubated with anti-CD16/32 (2.4G2, BD Biosciences, San Jose, CA, USA) to block nonspecific binding. Immunostaining was done in the presence of APC-CD25, FITC-CD8a, PE-CD4, PerCP-CD69, PE-Vio770-CD3ε (BD Biosciences, Biolegend or Miltenyi) at 4 °C for 30 min protected from light, and followed by two washes.

Cells were analyzed on the MACSQuant Analyzer (Miltenyi). Analyses of the multivariate data were performed using FlowJo v10 software (FlowJo, Ashland, OR, USA). The percent of CD8+ and CD3+ cells that were also positive for CD25 (or CD69) expression were based on data collected with greater than 50,000 cells from each sample.

### 4.10. B16F10 Syngeneic Tumor Model

FcγR-hu mice (8–10 weeks old female) were used for the syngeneic mouse lung tumor model. B16F10 tumor cells were intravenously injected into tail vein at 2 × 10^5^ cells/mouse, 0.2 mL of 1 × 10^6^ cells/mL in PBS [44,45]. At 30–60 min post IV injection, 0.2 mL of the test antibody sample was injected into the peritoneum. Antibody doses were given on day 0, 2, 4, 7, 9 and 11. On day 21, the mice were sacrificed and the lungs were weighed and scored for the number of metastases. The lung tumor index was determined by lung weight and tumor grade [68].

Tumor index = lung weight × grade for animal tumor

Grading:

1. Less than 10 tumor foci
2. 10–100 tumor foci
2.5. More than 100 foci, but countable
3. One lobe of the lung is full of tumor
4. Both lobes are full of tumor
5. Lungs are full of tumor and tumor growing out into cavities

### 4.11. Pharmacokinetics (PK)

For mouse antibody PK studies, female Tg32 hemi mice were injected with test antibody intravenously via tail vein at a dose of 2 mg/kg into 3 or 4 animals per group. Serial retro-orbital bleeds were obtained from CO_2_-anesthesized mice at indicated time points and terminal bleed was taken by cardiac puncture. After 30 min at RT, blood samples were centrifuged at 2500 rpm for 15 min and serum collected for analyses. All PK studies were approved by the Institutional Animal Care and Use Committee at Janssen Research & Development, LLC (Spring House, PA, USA).

An electrochemiluminescent immunoassay was used to measure human antibody concentration in mouse sera. Briefly, Streptavidin Gold multiarray 96-well plates 96-well plates (Meso Scale Discovery, Rockville, MD, USA) were coated with 50 μL/well of 1 μg/mL biotinylated F(ab′)_2_ goat anti-human IgG (H + L, Jackson Immunochemical) in Starting Block T20 (Thermo Scientific) overnight at 4 °C and washed with Tris-buffered saline with 0.05% (*w*/*v*) Tween 20 (TBST from Sigma). Standards and serum samples were prepared in sample buffer (1% (*w*/*v*) bovine serum albumin in TBST and 20 mM EDTA) added to plates and incubated for 2 h at RT on a shaker. Plates were washed and incubated for 1 h with 1 μg/mL MSD-Sulfo (ruthenium)-labeled with a pan huIgG1 Ab, R10Z8E9. Plates were washed, 1× Read buffer T was added and plates were read on the MSD Sector Imager 6000 (Meso Scale Discovery).

Terminal half-life (t_1/2_) calculations of the elimination phase for PK studies were determined using a 1-phase exponential decay model fitted by linear regression of natural log concentration versus time using Prism version 6.0 software. The least squares nonlinear decay model was weighted by 1/fitted concentration. Half-life calculations of the elimination phase were determined using the formula t_1/2_= −ln2/β, where β is the slope of the line fitted by the least square regression analysis starting after the first dose.

Monkey PK studies were performed at WuXi Apptec in China. Three animals were IV injected with test antibody at 1.5 mg/kg, and 1 mL of blood was collected via a cephalic vein at pre-dose, and at day 1, day 3, day 5, day 8, day 15 and day 22 post-dose. Serum samples were prepared and PK analyses were performed at Frontage (Shanghai) using a similar MSD format. Protocols were reviewed and approved by WuXi AppTec Institutional Animal Care and Use Committee (IACUC) prior to procedures (MGMT-011; TECH-030).

### 4.12. ImmunoFilter^TM^ Analyses

The amino acid sequences for the different variants were analyzed in silico using ImmunoFilter^TM^, an HLA class II-peptide binding prediction tool to predict comparative immunogenicity in humans (v2.7, Xencor, Inc., Monrovia, CA, USA, examples [69,70,71]). This prediction tool uses an immunochemical data set of peptide agretope binding to class II major histocompatibility complex (MHC) which can assess potential immunogenicity for more than 95% of U.S. population, based on empirical binding data. Output provided includes raw binding scores for peptides across a sequence of interest, standardized binding scores, binding probabilities to each allelic combination, and summary *IScores*, which are weighted, population-relevant values. For analyses, peptides from the wild-type and variant sequences with 100% identity to each other were excluded by application of a tolerance threshold, and only peptides spanning the sequences of interest were included. Resulting *IScores* were averaged across all loci, and plotted. Higher *IScores* indicate a higher predicted immunogenic risk (PIR).

### 4.13. Developability 

To assess whether antibodies with silent Fc regions would have good manufacturing properties, biophysical analytical tests were performed for thermal stability and solubility.

#### 4.13.1. Differential Scanning Calorimetry (DSC)

DSC experiments were performed using a MicroCal Auto VP-capillary DSC system (Malvern Instruments Ltd., Malvern, UK) in which temperature differences between the reference and sample cell were continuously measured and converted to power units. Samples were heated from 25 °C to 110 °C at a rate of 1 °C/min. A pre-scan time of 10 min and a filtering period of 10 s were used for each run. DSC measurements were made at sample concentrations of approximately 0.5 mg/mL in 1× PBS buffer in duplicate. Analysis of the resulting data was performed using MicroCal Origin 7 software (MicroCal, Northampton, MA, USA).

#### 4.13.2. Concentration Assessment

Antibody samples were concentrated by centrifugation at 2250× *g* using Amicon Ultra-15 centrifugal filter units with Ultracel-30 membranes (Sigma-Aldrich). Samples were inspected for signs of precipitation until volumes were reached for a concentration of 40–50 mg/mL. The sequence-predicted absorbance constants (A280/mg/mL) for each antibody were used to calculate sample concentrations at absorbance 280 nm.

#### 4.13.3. Dynamic Light Scattering (DLS)

Particle size and size distributions were determined using DLS on a DynaPro Plate Reader (Wyatt Technologies Corporation, Santa Barbara, CA, USA) at 23 °C. For each analysis, 30 μL of each sample at 1 mg/mL was placed in a 384-well black polystyrene plate with a clear flat bottom (Corning, CLS3540). Triplicate measurements were performed for each sample with each measurement consisting of 20 runs.

#### 4.13.4. Static Light Scattering (SLS), Thermal Melting (Tm) and Thermal Aggregation (Tagg) Analyses

Tm was determined for each sample using intrinsic fluorescence with the Uncle instrument (Unchained Labs, Pleasanton, CA, USA). Tagg was assessed by Static Light Scattering (SLS) to monitor protein aggregation using the same instrument. For the combined Tm and Tagg method, antibody was loaded and run with a thermal ramp from 15 to 95 °C; at a ramp rate of 0.3 °C/min.

#### 4.13.5. Size Exclusion Chromatography (SEC)

Samples were separated over a TOSOH TSKgel BioAssist G3SWxL column (7.8 mm × 30 cm, 5 μm, TOSOH) that had been equilibrated with PBS supplemented with 500 mM NaCl, at a flow rate of 0.5 mL/min using an Agilent 1100-series HPLC (Agilent Technologies, Santa Clara, CA, USA). A target of 100–200 μg of total protein was injected per run. Peaks were monitored using absorbance at 280 nm. Data analysis of species found in each sample was performed using ChemStation software (Agilent Technologies).

#### 4.13.6. Low pH Treatment 

Exposure to low pH was performed for accelerated stability testing. Protein samples were prepared at a concentration of 1 mg/mL. Samples were dialyzed for 6 h into 0.05 M sodium acetate buffer, pH 3.5; then dialyzed for 16 h in 0.1 M PBS, pH 7.4, and stored at 4 °C prior to analyses.

### 4.14. Crystallography

Recombinant huIgG1σ Fc and huIgG4σ2 Fc were transiently expressed in HEK 293 cells and purified by Protein A affinity chromatography at Sino Biological Inc. (China). Recombinant huIgG4σ1 Fc was transiently expressed in 293 Expi cells and purified in two steps using Protein A affinity chromatography and size exclusion chromatography (Superdex 200 PG) at Aldevron (Fargo, ND, USA). Proteins were delivered in 20 mM Tris, 50 mM NaCl, pH 7.5 at concentrations ranging from 2 to 4 mg/mL. Fc molecules were further concentrated to 10–13 mg/mL prior to crystallization. Crystallization experiments employed the vapor-diffusion method. Crystallization drops were set up using a Mosquito liquid handling robot (TTP Labtech, Melbourn, UK) in 96-well Corning 3550 (huIgG1σ and huIgG4σ2) or MRC 2 well (huIgG4σ1) crystallization plates. Diffraction quality crystal were obtained for all three Fc regions in 9–10% (*w*/*v*) PEG 20,000, 0.1 M sodium acetate, pH 5.5 (reservoir condition for huIgG4σ2 additionally contained 5% (*w*/*v*) MPD). Prior to data collection, crystals were cryo-protected in reservoir supplemented with 20% (*w*/*v*) glycerol and flash frozen in liquid N_2_. X-ray diffraction data for huIgG1σ and huIgG4σ1 were collected on the IMCA-CAT beam line (17-ID) at the Advanced Photon Source (APS) at Argonne National Laboratory equipped with a DECTRIS Pilatus 6M pixel array detector. Diffraction data for huIgG4σ2 were collected by Shamrock Structures, LLC on the SER-CAT beam line (22-ID) at APS equipped with a Rayonix 300HS CCD detector. All data were processed with the program XDS [72].

Initial phases were determined by the method of molecular replacement with the program Phaser [73] as implemented in the CCP4 suite of programs [74]. Individual C_H_2 and C_H_3 domains isolated from chain A of PDB 3AVE [40] (huIgG1 Fc) were provided as search models for huIgG1σ; and for huIgG4σ1 and huIgG4σ1, individual C_H_2 and C_H_3 domains were used from a previously refined internal structure of wild-type huIgG4 Fc. Phaser positioned the equivalent of one Fc dimer in the asymmetric unit in space group P2_1_2_1_2_1_. The structures underwent rounds of rebuilding and refinement with the programs Crystallographic Object-Oriented Toolkit (COOT) [75] and Phenix [76,77] respectively. Data collection and refinement statistics are provided in Table 2. The atomic coordinates and structure factors are archived in the Protein Data Bank under the accession numbers 5W5L, 5W5M, and 5W5N corresponding to huIgG1σ Fc, huIgG4 σ1 Fc, and huIgG4 σ2 Fc, respectively.

### 4.15. Molecular Dynamics Simulations

Explicit solvent MD simulations were performed on intact Fc to investigate the flexibility of BC and FG loops. Two simulations were initiated from the available crystal structures: IgG1 WT using the crystal structure (PDB 3AVE) of IgG1 wild-type Fc and IgG1σ using the crystal structure described here. Additional two simulations were initiated after modelling mutations in the wild-type crystal structure: IgG1-H268A containing H268A mutation and IgG1-SS containing A330S and P331S mutations. The simulations included both chains of C_H_2 and C_H_3 domains, and the glycans present in the corresponding crystal structures. The simulations were set up in the Maestro graphical user interface and run using the Desmond program (multisim version 3.8.5.19 and mmshare version 3.5) [78], both part of the Schrodinger 2016-3 suite [79]. The systems were protonated at neutral pH and centered in an orthorhombic box such that the minimum distance from any protein atom to the box wall was 10 Å. The box was solvated using SPC [80] water molecules and counter ions were added to neutralize the system. OPLS3 force field [81] was used as the potential energy function for the protein. Replica Exchange Solute Tempering [82] (REST) MD simulations were performed at 300 K and 1 bar using 16 replicas with the BC (residue numbers 267–273) and FG (residue numbers 322–333) residues of only chain B specified as “hot”. REST MD simulations were designed to enhance sampling of the hot subset of the full simulation system. Note that sampling is enhanced only in chain B since chain A loops are cold. To get an idea of the degree by which sampling is enhanced by REST, we compared the backbone fluctuations of the two chains in the C_H_2 domain. Figure S7 shows that it was advantageous to use the REST approach for all systems except IgG1σ. Default implementation of REST in Desmond was used for initial equilibration, setting up energy function of each replica, specifying exchange protocol and recording trajectory data. Simulations were performed on AWS (Amazon Web Services) cloud computing platform with each simulation employing 8 NVIDIA Tesla K80 GPU cards (Nvidia, Santa Clara, CA, USA). The production run was 100 nanoseconds long and the final trajectory from each replica contained 4166 conformations saved at an interval of 24 ps. Number of atoms in the simulations was approximately 53,000 and a single simulation took approximately 74 hours. A time step of 2 femtoseconds was used and exchanges were attempted at an interval of 1.2 picoseconds. In all simulations, the acceptance ratio of exchanges between the adjacent replicas was observed to be between 0.2 and 0.4. The results presented here correspond to the trajectory from the physical replica for which the energy function is unperturbed.

## 5. Conclusions

A requirement for truly silent human Fc designs is evident as more antibodies are being developed for immunotherapy. The Fc engineered human mutations described here as IgG1σ, IgG4σ1 and IgG4σ2 demonstrate equal or lower immune functionality than the corresponding IgG2σ design. Lack of immune effector function is demonstrated in vitro with FcγR binding, cytotoxicity assays, and in vivo with T cell activation and tumor inhibition studies. Crystal structures and simulations of these Fc variants reveal altered conformational preferences within the lower hinge and BC and FG loops relative to wild-type IgG, providing a structural rationalization for diminished Fc receptor engagement. Immunogenicity predictions, pharmacokinetic studies in mice and monkeys, and biophysical analyses also support these novel mutations as optimized silent Fc choices for the development of therapeutic antibodies. 

## Figures and Tables

**Table 1:** List of mutations in the hinge and constant heavy chain domain 2 (C_H_2) regions of different silent immunoglobulin (IgG) designs.

	IgG1 AA	IgG1σ	IgG1 FES	IgG2σ	IgG4 PAA	IgG4σ1	IgG4σ2
**Core hinge**					S228P	S228P	S228P
**Lower hinge**	L234A	L234A	L234F	V234A	F234A	F234A	F234A
	L235A	L235A	L235E		L235A	L235A	L235A
							∆G236
		G237A		G237A		G237A	G237A
		P238S		P238S		P238S	P238S
**C_H_2 domain of Fc**		H268A		H268A			
				V309L			
		A330S		A330S			
		P331S	P331S	P331S			

Δ Indicates deletion of residue. European Union (EU) numbering is used for the amino acid substitutions.

**Table 2:** Thermal stability of the Fc variant antibody panel.

Ab	Fc Variant	Differential Scanning Calorimetry Data			Colloidal Scattering at Two Wavelengths	
		Tm1 (°C)	Tm2 (°C)	Tm3 (°C)	Tagg_266nm_ (°C)	Tagg_473nm_ (°C)
Ab1	IgG1	71.7 ± 0.1	75.2 ± 0.1	82.6 ± 0.1	67.7 ± 0.6	69.7 ± 0.7
Ab1	IgG1 AA	71.7 ± 0.2	75.0 ± 0.1	82.2 ± 0.1	68.0 ± 0.3	69.9 ± 0.5
Ab1	IgG1 FES	64.7 ± 0.0	74.0 ± 0.1	82.5 ± 0.0	ND	ND
Ab1	IgG1σ	60.8 ± 0.1	74.4 ± 0.1	82.7 ± 0.1	68.3 ± 0.7	69.8 ± 0.6
Ab1	IgG2	71.6 ± 0.1	75.9 ± 0.1	ND	65.3 ± 0.3	67.6 ± 0.3
Ab1	IgG2σ	62.0 ± 0.2	74.5 ± 0.1	71.4 ± 0.2	64.0 ± 2.1	66.0 ± 1.7
Ab1	IgG4 PAA	69.5 ± 0.1	73.4 ± 0.1	ND	63.8 ± 1.7	66.0 ± 0.6
Ab1	IgG4σ1	62.0 ± 0.2	70.8 ± 0.1	73.8 ± 0.1	64.1 ± 1.4	66.0 ± 0.5
Ab1	IgG4σ2	61.2 ± 0.6	69.9 ± 0.6	73.2 ± 0.4	63.8 ± 1.8	66.0 ± 0.5

Antibody (Ab) samples are in PBS; values are shown as ± range. Abbreviations: ND, no data; fragment crystallizable, Fc; temperature of melting at midpoint, Tm; IgG1 AA, IgG1 L234A/L235A; IgG1 FES, IgG1 L234F/L235E/P331S; IgG4 PAA, IgG4 S228P/F234A/L235A.

**Table 3:** Stability of Concentrated Proteins Stored at 4 °C (concentration of antibodies over time).

Sample ID	Release concentration (mg/mL)	Week 1 (mg/mL)	Week 2 (mg/mL)	Week 3 (mg/mL)	Week 4 (mg/mL)
IgG1	53.9 ± 0.8	53.1 ± 0.3	57.2 ± 0.7	55.1 ± 0.4	55.0 ± 0.3
IgG1 FES	45.5 ± 0.9	48.8 ± 1.2	46.4 ± 0.7	46.7 ± 0.3	46.6 ± 1.5
IgG1σ	41.9 ± 0.6	41.5 ± 0.2	42.0 ± 0.3	42.5 ± 0.9	43.4 ± 0.5
IgG2	41.4 ± 0.1	40.5 ± 0.2	42.0 ± 0.4	42.9 ± 0.2	43.0 ± 0.8
IgG2σ	40.8 ± 0.5	41.3 ± 0.4	40.9 ± 0.6	41.7 ± 1.1	41.8 ± 0.6
IgG4-PAA	48.6 ± 1.5	50.6 ± 0.6	52.5 ± 1.0	53.2 ± 0.2	50.9 ± 1.3
IgG4σ1	50.4 ± 0.4	50.3 ± 0.1	50.0 ± 0.6	50.0 ± 0.2	51.4 ± 0.8
IgG4σ2	51.7 ± 1.1	51.3 ± 0.4	48.4 ± 2.1	53.4 ± 1.6	54.8 ± 0.7

Ab samples are in PBS and shown as mean ± range. Abbreviations: IgG1 AA, IgG1 L234A/L235A; IgG1 FES, IgG1 L234F/L235E/P331S; IgG4 PAA, IgG4 S228P/F234A/L235A.

**Table 4:** Size exclusion chromatography (SEC) analyses with percent monomer of concentrated proteins.

Sample Name	Release (%)	Week 1 (%)	Week 2 (%)	Week 4 (%)
IgG1	100.0	100.0	99.6	99.4
IgG1 FES	100.0	100.0	100.0	99.0
IgG1σ	100.0	100.0	99.7	99.3
IgG2	98.17	98.0	97.9	97.7
IgG2σ	100.0	100.0	99.2	100.0
IgG4 PAA	100.0	99.6	99.5	99.5
IgG4σ1	98.0	97.9	97.7	97.5
IgG4σ2	97.8	97.9	97.7	97.4

Representative data of SEC analysis. Abbreviations: IgG1 AA, IgG1 L234A/L235A; IgG1 FES, IgG1 L234F/L235E/P331S; IgG4 PAA, IgG4 S228P/F234A/L235A.

**Table 5:** Tm and temperature of aggregation (Tagg) of Fc variants at high concentration (40–50 mg/mL).

Antibody	Week	Tm1 (°C)	Tagg_266nm_ (°C)
IgG1	R	69.3 ± 1.4	68.0 ± 0.1
IgG1	Week 0	68.2 ± 0.5	67.4 ± 0.6
IgG1	Week 4	68.3 ± 0.3	67.6 ± 0.8
IgG1σ	R	61.5 ± 1.0	68.9 ± 0.3
IgG1σ	Week 0	60.6 ± 0.2	67.8 ± 0.3
IgG1σ	Week 4	60.8 ± 0.1	67.9 ± 0.7
IgG1 AA	R	69.3 ± 0.5	68.3 ± 0.3
IgG1 AA	Week 0	68.2 ± 0.0	67.7 ± 0.2
IgG1 AA	Week 4	68.4 ± 0.1	68.1 ± 0.2
IgG1 FES	R	65.9 ± 0.8	67.7 ± 0.1
IgG1 FES	Week 0	64.9 ± 0.6	66.9 ± 0.3
IgG1 FES	Week 4	65.1 ± 0.1	67.5 ± 0.2
IgG2	R	67.5 ± 1.8	65.1 ± 0.8
IgG2	Week 0	66.5 ± 0.4	65.6 ± 0.1
IgG2	Week 4	66.7 ± 0.4	65.2 ± 1
IgG2σ	R	61.7 ± 0.8	61.9 ± 2.4
IgG2σ	Week 0	62.6 ± 0.2	66.2 ± 0.1
IgG2σ	Week 4	62.7 ± 0.2	66.8 ± 0.4
IgG4 PAA	R	66.4 ± 1.9	62.1 ± 0.5
IgG4 PAA	Week 0	66.2 ± 0.6	65.5 ± 1.3
IgG4 PAA	Week 4	66.3 ± 0.2	65.9 ± 0.1
IgG4σ1	R	61.5 ± 0.1	62.7 ± 0.9
IgG4σ1	Week 0	62.0 ± 0.2	65.5 ± 0.2
IgG4σ1	Week 4	62.0 ± 0.4	66.1 ± 0.0
IgG4σ2	R	61.6 ± 0.7	62.0 ± 1.5
IgG4σ2	Week 0	61.8 ± 0.1	65.6 ± 0.1
IgG4σ2	Week 4	62.1 ± 0.2	65.9 ± 0.1

Tm1 and Tagg_266nm_ values = mean ± range; Ab samples are in PBS. R is the Release material; Week 0 is data after concentration of material; Week 4 is data after week 4 of incubation.

**Table 6:** Dynamic light scattering data after low pH treatment.

Ab1	Avg R_h_	Avg %Pd	Avg %Mass	Avg R_h_	Avg %Pd	Avg %Mass
	Release Material			Low pH Material		
IgG1	5.4 ± 0.1	10.3 ± 2.1	100.0 ± 0.0	5.1 ± 0.2	12.2 ± 1.7	100.0 ± 0.0
IgG1σ	5.4 ± 0.0	9.6 ± 0.3	100.0 ± 0.0	5.0 ± 0.2	13.2 ± 0.8	100.0 ± 0.0
IgG1 AA	5.8 ± 0.3	16.7 ± 10.1	100.0 ± 0.1	5.5 ± 0.2	12.2 ± 7.1	100.0 ± 0.0
IgG1 FES	5.5 ± 0.0	7.7 ± 1.6	100 ± 0.0	5.2 ± 0.1	11.9 ± 1.3	99.7 ± 0.6
IgG2	5.8 ± 0.1	18.2 ± 5.2	99.6 ± 0.1	5.0 ± 0.1	13.2 ± 0.6	100.0 ± 0.0
IgG2σ	5.4 ± 0.0	13.0 ± 3.6	100.0 ± 0.0	5.1 ± 0.2	12.5 ± 1.5	99.6 ± 0.8
IgG4—PAA	5.3 ± 0.1	9.9 ± 0.6	100.0 ± 0.0	5.3 ± 0.0	10.4 ± 0.8	100.0 ± 0.0
IgG4σ1	5.4 ± 0.1	9.3 ± 0.5	100.0 ± 0.0	6.4 ± 0.2	15.5 ± 1.6	100.0 ± 0.0
IgG4σ2	5.4 ± 0.0	8.9 ± 0.4	100.0 ± 0.0	6.6 ± 0.1	14.8 ± 1.3	100.0 ± 0.0

Samples were tested at 1 mg/mL in triplicate and shown as mean ± standard deviation (STD). Avg—average; Percent polydispersity (% Pd); hydrodynamic radius (R_h_).

**Table 7:** X-ray crystallographic data and refinement statistics.

	IgG1σ Fc	IgG4σ1 Fc	IgG4σ2 Fc
**Crystal Data**			
Space group	P2_1_2_1_2_1_	P2_1_2_1_2_1_	P2_1_2_1_2_1_
Unit cell parameters			
a, b, c (Å)	73.36, 79.17, 101.44	74.74, 78.39, 97.39	74.52, 78.47, 97.51
α, β, γ (°)	90.0, 90.0, 90.0	90.0, 90.0, 90.0	90.0, 90.0, 90.0
Resolution (Å)	50.00–1.90 (1.95–1.90) ^a^	50.00–1.90 (1.95–1.90)	50.0–1.85 (1.90–1.85)
Measured reflections	256,516 (19,366)	248,644 (18,842)	363,451 (27,057)
Unique reflections	47,175 (3427)	45,491 (3348)	49,481 (3648)
Completeness (%)	99.7 (99.7)	99.4 (99.9)	99.9 (100.0)
Redundancy	5.4 (5.7)	5.5 (5.6)	7.3 (7.4)
R_merge_^b^	0.041 (0.539)	0.045 (0.588)	0.056 (0.670)
<I/σ> ^c^	21.3 (2.9)	18.6 (2.8)	19.8 (3.0)
**Refinement Statistics**			
Resolution (Å)	33.28–1.90	32.70–1.90	47.26–1.85
Number of reflections	47,088	45,473	49,469
R_work_ (%) ^d^	19.2	18.2	18.1
R_free_ (%) ^d^	22.9	22.5	21.5
Number atoms	3772	3771	3897
Protein	3292	3320	3404
Carbohydrate	220	220	210
Solvent	260	231	283
Mean B-factor (Å^2^)	41.9	45.9	40.0
Protein	40.4	43.7	37.9
Carbohydrate	58.5	75.7	66.2
Solvent	46.4	50.0	45.9
RMSD ^e^			
Bond lengths (Å)	0.009	0.012	0.018
Bond angles (°)	1.213	1.438	1.699
Ramachandran			
Favored (%)	98.1	98.8	98.1
Allowed (%)	1.9	1.2	1.9
Outliers (%)	0.0	0.0	0.0

^a^ Values for high resolution shell are shown in parentheses. ^b^ R_merge_ = Σ_hkl_Σ_i_(|<I_hkl_> − I_hkl,i_|)/Σ_hkl_Σ_i_ I_hkl,I_, where hkl enumerates the unique reflections and i their symmetry-equivalent or multiply-measured contributors. I is the intensity for a given measurement. ^c^ <I/σ> is the average x-ray reflection intensity (I) measurement divided by the standard deviation of that measurement (σ) for the whole X-ray data set. ^d^ R = (Σ_hkl_ ||F_obs_(hkl)| – |F_calc_(hkl)||)/(Σ_hkl_ |F_obs_(hkl)|), where F_obs_(hkl) and F_calc_(hkl) are the observed and calculated structure factors. R_work_ includes structure factors for the entire data set minus those data sequestered for computing R_free_. R_free_ includes only the latter randomly chosen subset of the data. ^e^ RMSD (Root Mean Square Deviation) in the table describes how well the atomic bond distances and angles compare to idealized values determined from a set of reference structures.

**Table 8:** Summary of Silent Fc Designs.

	IgG1 WT	IgG1 AA	IgG1σ	IgG1 FES	IgG2σ	IgG4 PAA	IgG4σ1	IgG4σ2
Number of Mutations	0	2	7	3	7	3	5	6
Lack of Fc Immune Functions ^1^	+	++/+	++++	++/+++	++++	+++	++++	++++
Developability ^2^	++++	++++	++++	++++	+++	++++	++++	++++
PK ^3^	++++	+++	++++	++++	+++	++++	++++	++++
Low Immunogenicity Risk ^4^ (*in silico*)	++++	++++	++++	ND	+++	++++	++++	+++
Low PBMC Immunogenicity ^5^	++++	++++	ND	ND	++++	++++	ND	ND

ND indicates no data. ^1^ Lack of Fc Immune Functions was determined collectively by FcγR binding, ADCC, ADCP, CDC, in vivo T cell activation, and *in vivo* tumor cell inhibition studies. The least silent is denoted by + and ranges to ++++ as the most silent. ^2^ Developability was determined by thermal stability, stability of concentrated IgG samples and stability after low pH treatment. Good is +, intermediate is +++, and best is ++++. ^3^ PK was performed in human FcRn transgenic mice and in cynomolgus monkeys. Variants with longer half-life are denoted ++++, and ones with slightly shorter half-life are shown as +++. ^4^ Low Immunogenicity Risk was predicted using a T cell epitope analyses software. ++++ indicates low risk and +++ is medium to low risk. ^5^ Low PBMC immunogenicity data from a previous report are included because huIgG1σ and huIgG4σ variants have very similar sequences. ++++ indicates low risk.

## Supplementary Materials

The following are available online at www.mdpi.com/2073-4468/6/3/12/s1; Table S1: Cynomolgus monkey PK parameters for Ab2. Figure S1: Electron density map about lower hinge residues, Figure S2: Altered lower hinge conformation of huIgG1σ is not a requirement of crystal packing, Figure S3: Crystal packing does not prevent huIgG1σ FG loop from adopting a flipped-out conformation, Figure S4: Conformation distribution of P329, Figure S5: Conformation distribution of P271, Figure S6: Interaction of lower hinge and FG loop with FcγR, Figure S7: REST MD enhances loop sampling.
