# Supplementary material for: Functional, Biophysical, and Structural Characterization of Human IgG1 and IgG4 Fc Variants with Ablated Immune Functionality
Source: Antibodies (Basel). 2017 Sep 1;6(3):12. doi: 10.3390/antib6030012 (PMC6698826; doi:10.3390/antib6030012)
Supplement: Supplementary file 1 [file antibodies-06-00012-s001.pdf]

Supplementary Materials:

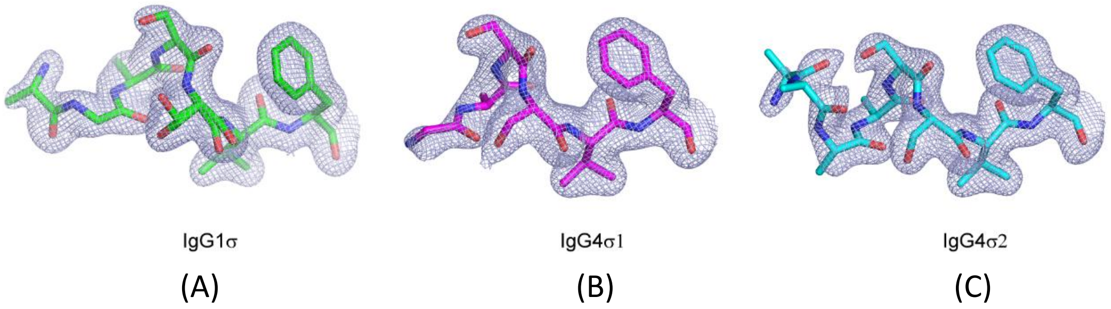

Figure S1. Electron density map about lower hinge residues. 2mFo-DFc density contoured at 1σ is shown carved at a distance of 1.5 Å about IgG1σ residues A235-F241 (**left**, green stick), IgG4 σ1 residues G236-F241 (**middle**, magenta stick), and IgG4 σ2 residues E233-F241 (**right**, cyan stick) from chain B.

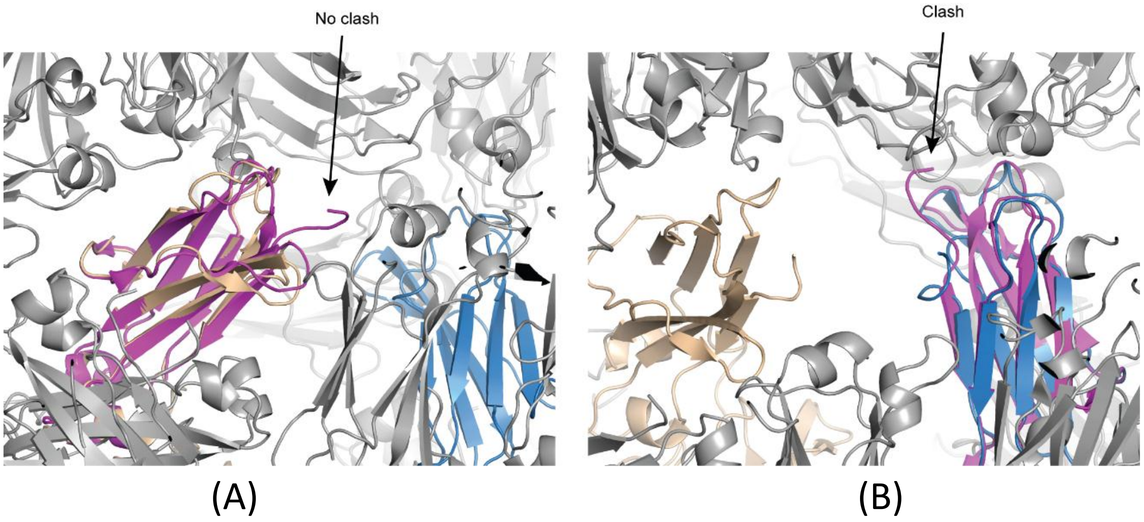

Figure S2. Altered lower hinge conformation of huIgG1σ is not a requirement of crystal packing. CH2 domain from wild-type huIgG1 (3AVE [40], chain A, magenta cartoon) is aligned to CH2 domain from huIgG1σ chain A (**left**, wheat cartoon) and chain B (**right**, sky blue cartoon). Symmetry related copies of huIgG1σ Fc are shown in the grey cartoon.

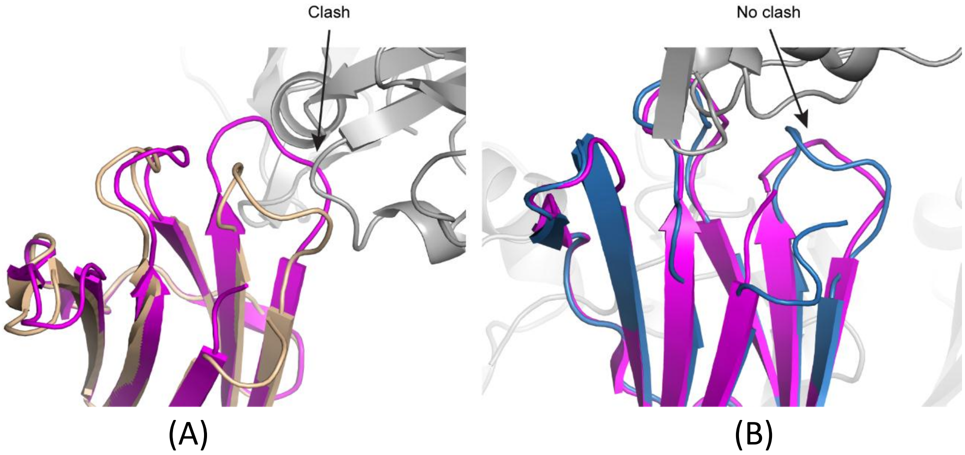

Figure S3. Crystal packing does not prevent IgG1σ FG loop from adopting a flipped-out conformation. CH2 domain from huIgG2σ (4L4J [29], chain A, magenta cartoon) is aligned to CH2 domain from huIgG1σ chain A (**left**, wheat cartoon) and chain B (**right**, sky blue cartoon). Symmetry related copies of huIgG1σ Fc are shown in the grey cartoon.

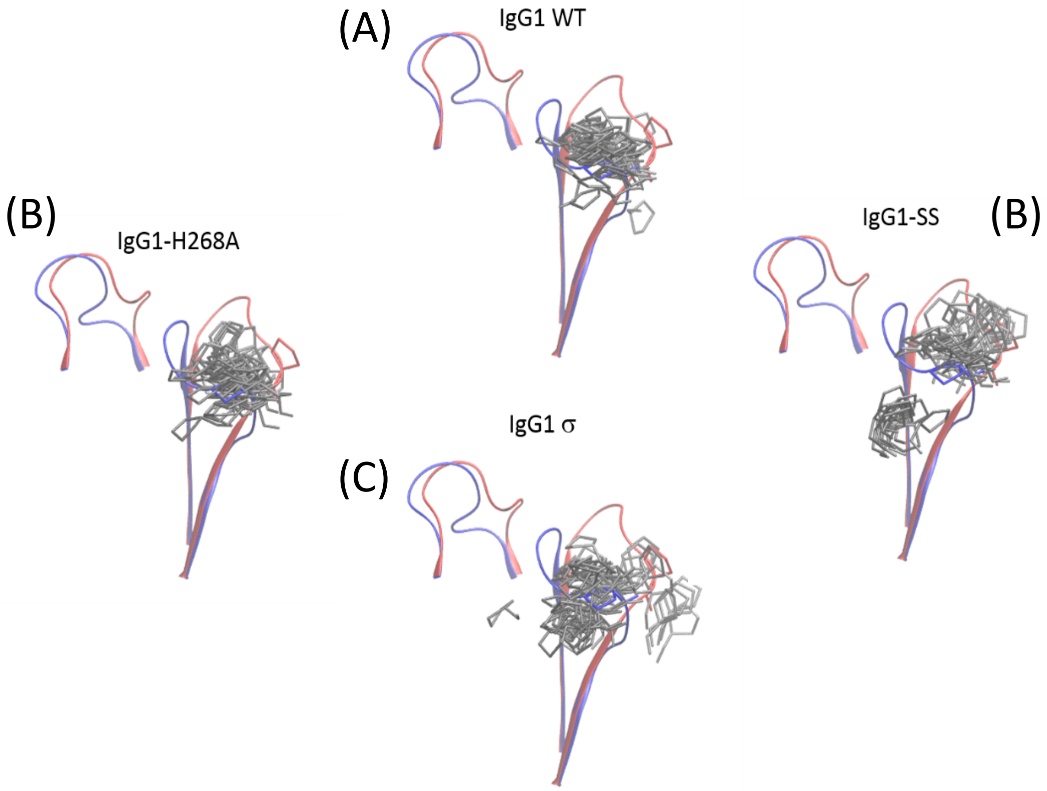

Figure S4. Conformation distribution of P329. Conformations of the non-hydrogen atoms of P329 in the fifty FG loop conformations shown in Figure 15.

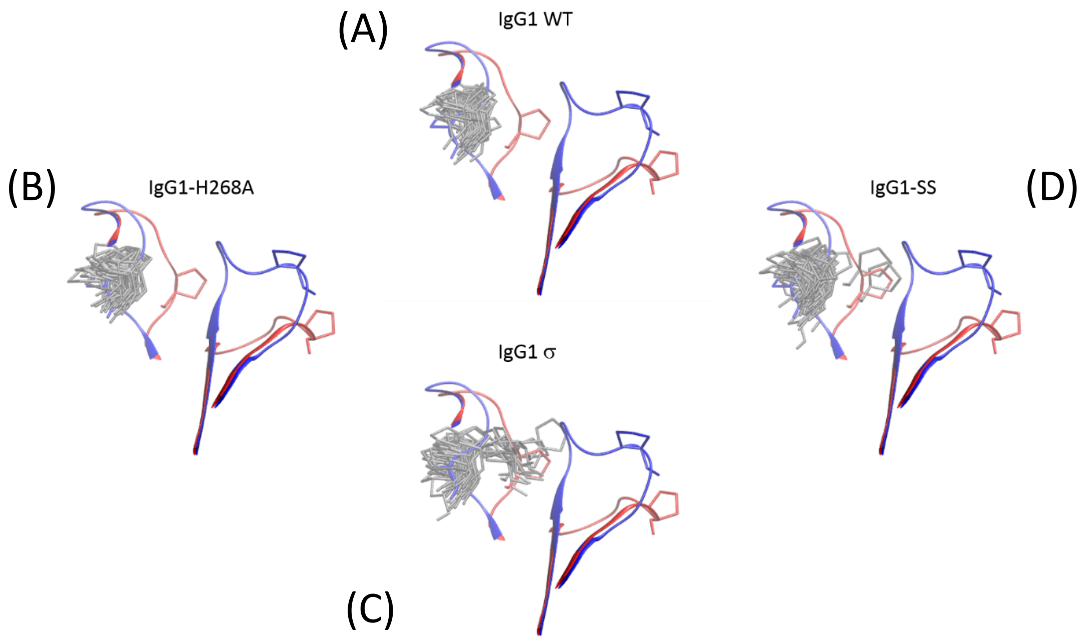

Figure S5. Conformation distribution of P271. Conformation of the non-hydrogen atoms of P271 in the fifty BC loop conformations shown in Figure 16.

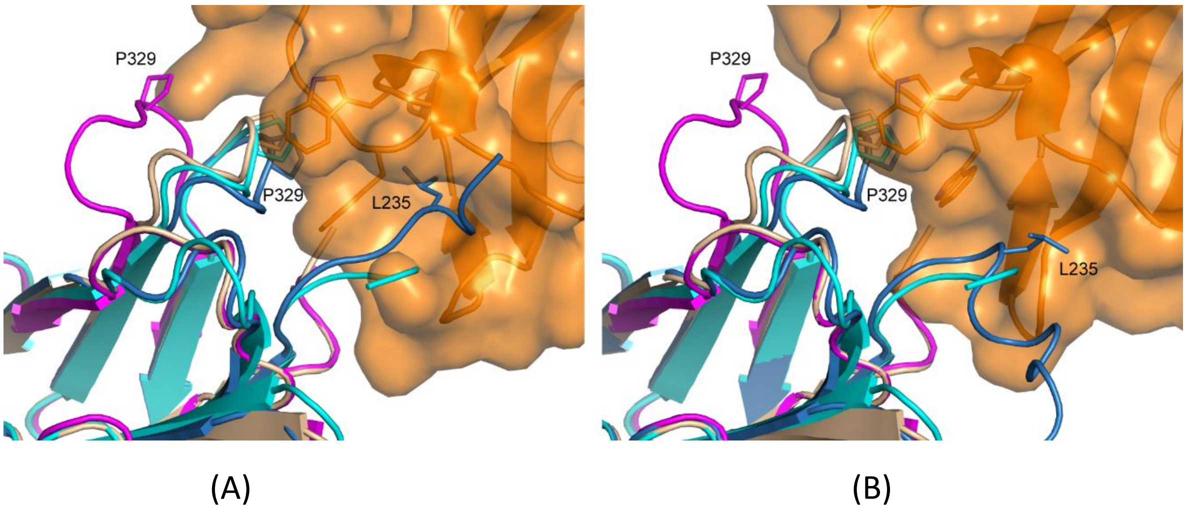

Figure S6. Interaction of lower hinge and FG loop with FcγR. Fc (blue, cartoon) is shown in complex with receptor (orange, cartoon and transparent surface) FcγRI (**left**) or FcγRIIIa (**right**) from PDBs 4W4O [51] and 3SGJ [47], respectively. Fc lower hinge residue L235 and FG loop residue P329 are shown in stick and labeled. CH2 domains from apo crystal structures of wild-type huIgG1 (3AVE chain A [40]; cyan cartoon), huIgG1σ Fc (chain B; wheat cartoon) and IgG4σ1 Fc (chain B: magenta cartoon) are shown aligned to the CH2 domain of the receptor bound Fc with residue P329 shown in stick, labeled.

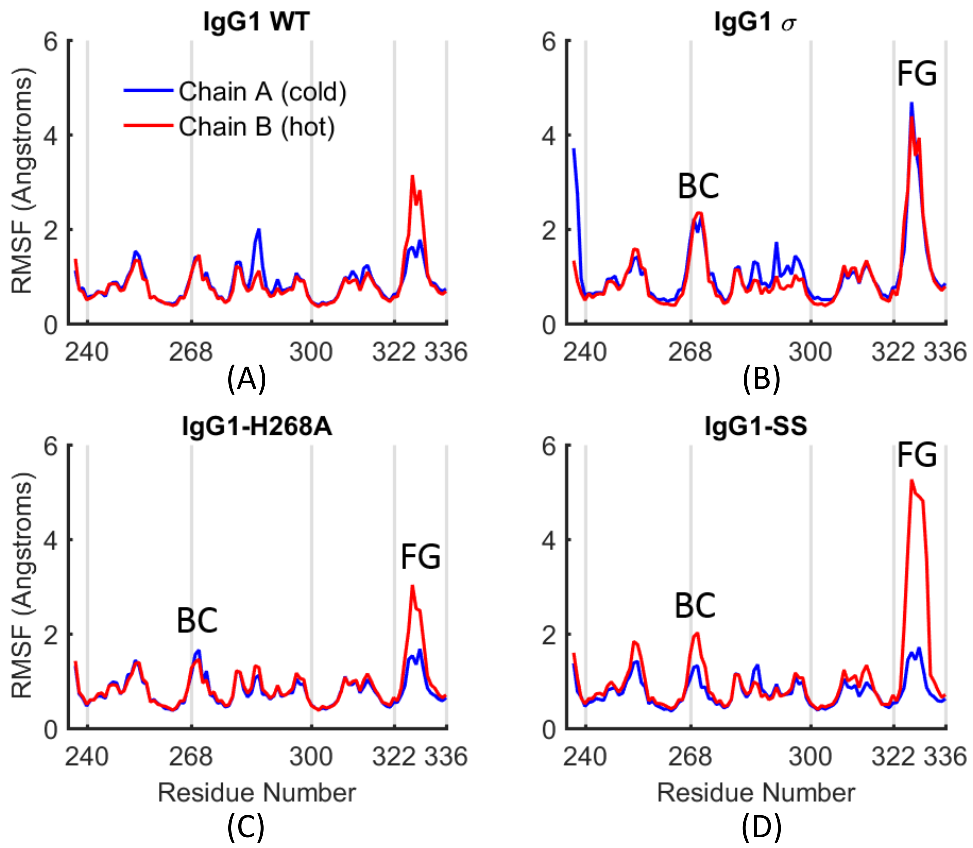

Figure S7. REST MD enhances loop sampling. Root mean squared fluctuations (RMSF) of the Cα atoms for the two chains of CH2 domain from molecular dynamics simulations. The REST MD simulations were designed to increase the sampling of the BC and FG loops of chain B which constituted the hot regions of the simulation; chain A (blue) was cold. RMSF for a given chain is calculated after aligning the trajectories on the Cα atoms of the non-BC and non-FG loop residues of that chain. REST MD substantially improves the sampling of the FG loop in all simulations except IgG1σ; impact on the sampling of the BC loop are relatively modest.

Table S1. Cynomolgus monkey PK parameters for Ab2.

Ab2 (IgG1 WT)*	Animals (F, M)	Dose (mg/kg)	Half-life ± SD (d)	CL + SD (mL/day/kg)
2009 study	3	1.5	11.8 ± 4.2	4.72 ± 1.06
2010 study	4	1.5	10.5 ± 0.9	4.57 ± 0.91
2014 study	5	0.5	11.7 ± 1.0	3.67 ± 1.26

* An anti-RSV antibody, similar to Ab2, has a human half-life of approximately 23 days.
